# Dual role of PID1 in regulating apoptosis induced by distinct anticancer-agents through AKT/Raf-1-dependent pathway in hepatocellular carcinoma

**DOI:** 10.1038/s41420-023-01405-1

**Published:** 2023-04-28

**Authors:** Jian Yang, Senlin Li, Jialuo He, Qianqian Xu, Mengyuan Xie, Ci Yang, Hongjie Wang, Yonghui Zhang, Qian Wan, Ming Xiang

**Affiliations:** 1 Department of Pharmacology, School of Pharmacy, Tongji Medical College, Huazhong University of Science and Technology, Wuhan, 430030 China; 2 Hubei Key Laboratory of Natural Medicinal Chemistry and Resource Evaluation, School of Pharmacy, Tongji Medical College, Huazhong University of Science and Technology, Wuhan, 430030 China; 3 Department of Virology, College of Life Sciences, Wuhan University, Wuhan, 430072 China; 4 Institute for Human Health & Disease Intervention, Department of Chemistry and Biochemistry, Florida Atlantic University, Jupiter, 33458 FL USA

**Keywords:** Oncogenes, Apoptosis, Growth factor signalling

## Abstract

The treatment outcome of hepatocellular carcinoma (HCC) is severely hampered due to its etiology, and thus in depth understanding of the genetic mechanisms underlying response of HCC to various anticancer agents is needed. Here, we have identified Phosphotyrosine interaction domain-containing protein 1 (PID1) as a novel regulator involved in modulation of apoptosis induced by anticancer agents in a context-dependent manner. PID1 relieved chemotherapy-induced ROS production, mitochondrial outer membrane permeability and mitochondrial respiratory depression. In addition, PID1 restricted AKT-mediated inhibition on Raf-1 through interacting with PDPK1 at phosphorylated tyrosine sites, thus enhancing Raf-1-mediated BAD inhibition. Interestingly, AKT, Bcl2 inhibition or Raf-1 silencing abolished PID1-mediated anti-apoptotic effects. However, PID1 altered the rhythmicity of pharmacological activity of Sorafenib on various survival-related kinases, thus resulting in AKT blockade via Raf-1/BRAF/ERK/MEK pathway. BRAF inhibition or Raf-1 depletion disrupted PID1-mediated barrier in AKT activation in response to Sorafenib. Moreover, in vivo study indicated that PID1 deficiency led to increased survival rate upon Doxorubicin treatment but reduced efficacy of Sorafenib. Overall, we propose that PID1 can function as an underlying biomarker of resistance to conventional chemotherapeutic agents but sensitivity towards Sorafenib.

## Introduction

Liver cancer remains to be one of the leading causes of cancer-related mortality, with an estimated incidence of 1> million cases by 2025, and hepatocellular carcinoma (HCC) accounts for 90% of these cases [1]. Over the past decades, numerous studies have been conducted to investigate the underlying mechanisms responsible for HCC development, and different therapeutic strategies for HCC have been developed, including surgical resection, radiofrequency ablation (RFA), trans-arterial chemoembolization (TACE) and systemic therapy. However, surgical resection and RFA are often inadequate because of the delayed diagnosis, resulting in widespread application of TACE and systemic therapy [2].

Sorafenib is a multi-kinase inhibitor approved as a first-line targeted therapy for the management of advanced HCC [3]. It can block proliferation and angiogenesis by inhibiting MAPK/ERK pathway as well as kinase activity of growth factor receptors such as VEGFR and PDGFR-β [3]. Conventional chemotherapeutic agents such as Doxorubicin and Cisplatin have been developed based on their capacity to inhibit DNA or enzymes required for DNA synthesis [4]. Although chemotherapeutic agents employed for systemic treatment have exhibited minimal success rate owing to their limited drug targeted ability, TACE can effectively solve this problem, leading to widespread application of conventional TACE for intermediate HCC [5]. Importantly, the efficiency of HCC treatment is severely hampered due to its etiology [6]. Hence, further understanding of the genetic mechanisms underlying response of HCC to diverse anticancer agents is required.

Phosphotyrosine interaction domain-containing protein 1 (PID1), an adaptor protein containing a phosphotyrosine binding (PTB) domain, was initially reported to be involved in development of insulin resistance via inhibiting PI3K/AKT pathway, and its primary function involved the regulation of proliferation and differentiation [7, 8]. Several studies have demonstrated that PID1 can regulate lipid metabolism in liver through interacting with LRP1 [9, 10]. Besides, PID1 could correlate with efficacy of chemotherapeutic agents in glioma, suggesting that PID1 could be implicated in modulation of various biological processes [11]. However, little is known about the role of PID1 in HCC development.

Activation of AKT or Raf-1 has been reported to be essential for HCC development [12, 13]. The function of Raf-1 is mainly regulated by phosphorylation at several sites. Phosphorylation at Ser338 and Tyr341 can lead to activation of Raf-1, whereas phosphorylation at Ser259 can inactivate Raf-1, thereby inhibiting MAPK/ERK pathway [14, 15]. The observation that p-AKT could directly phosphorylate Raf-1 at Ser259 suggests that AKT might also be involved in inhibiting Raf-1 [16]. Importantly, both AKT and Raf-1 activation can inhibit the function of Bcl2-associated agonist of cell death (BAD), unrestrained activity of which has been found to facilitate mitochondria-dependent apoptosis [17, 18]. Thus, fine-tuning of the balance between AKT and Raf-1 can potentially determine the efficiency of different anticancer agents. However, the mechanisms and regulators involved in this process are poorly understood.

Here, we describe the dual role of PID1 in regulation of apoptosis induced by distinct anticancer agents in HCC. It was found that on the one hand, PID1 relieved AKT-mediated inhibition on Raf-1 via interacting with PDPK1, thus facilitating Raf-1-mediated survival signals and inhibiting apoptosis induced by chemotherapeutic agents. On the other hand, PID1 accelerated Sorafenib-induced Raf-1 activation and promoted ERK-mediated feedback pathway, thereby blocking AKT activation and promoting apoptosis. These results reveal the novel function of PID1 in regulating apoptosis via modulating AKT/Raf-1-dependent pathway, and establish PID1 as a predictive biomarker of resistance to Doxorubicin but sensitivity to Sorafenib.

## Results

### PID1 levels predicted resistance to mitochondria-dependent apoptosis

**Fig. 1:**
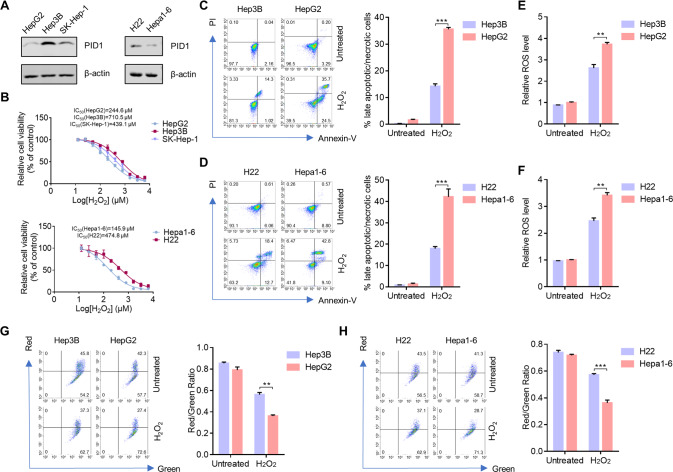
PID1 levels predicted resistance to mitochondria-dependent apoptosis. **A** Protein levels of PID1 in three human hepatoma cell lines (Hep3B, HepG2 and SK-Hep-1) and two mice hepatoma cell lines (H22, Hepa1–6). **B** Cell viability curves of three human hepatoma cell lines and two mice hepatoma cell lines treated with the indicated doses of H_2_O_2_ for 24 h. **C**–**D** Flow cytometry analysis measuring apoptosis in four hepatoma cell lines treated with H_2_O_2_ (200 μM) for 24 h. **E**–**F** H_2_DCFDA probe was used to detect intracellular ROS in four hepatoma cell lines treated with H_2_O_2_ (200 μM) for 24 h by flow cytometry analysis, and mean fluorescence intensity (MFI) was calculated. **G**–**H** JC-1 probe was used to detect mitochondrial membrane potential in four hepatoma cell lines treated with H_2_O_2_ (200 μM) for 24 h by flow cytometry analysis, and Red/Green Ratio was calculated. Data are expressed as mean ± SD (*n* = 3). **p < 0.01; ***p < 0.001.

We initially assessed the PID1 levels in three human hepatoma cell lines (HepG2, Hep3B and SK-Hep-1) and two mice hepatoma cell lines (Hepa1–6 and H22). Hep3B and H22 cells possess high PID1 expression, whereas HepG2 and Hepa1–6 cells possess lower PID1 levels (Fig. 1A). Hepatoma cells with high PID1 levels were more resistant to oxidative stress (Fig. 1B), and significantly higher percentage of apoptosis was detected in HepG2 and Hepa1–6 cells (Fig. 1C, D). Oxidative stress-induced mitochondrial dysfunctions, such as ROS burst and mitochondrial outer membrane permeability, are directly related to mitochondria-dependent apoptosis [19–21]. We then used H_2_DCFDA probe (a probe could be oxidized by ROS and yield fluorescence) to detect ROS levels and JC-1 probe (a probe undergoes shift of fluorescence from red to green once mitochondria get depolarized in apoptotic cells) to evaluate mitochondrial membrane potential (MMP). We subsequently observed that HepG2 and Hepa1–6 cells exhibited higher ROS level (Fig. 1E, F) but lower MMP (Fig. 1G, H) in response to H_2_O_2_ compared to Hep3B and H22 cells, suggesting that PID1 levels could predict the resistance to mitochondria-dependent apoptosis.

### PID1 reduced efficacy of chemotherapeutic agents but facilitated Sorafenib-induced apoptosis

**Fig. 2:**
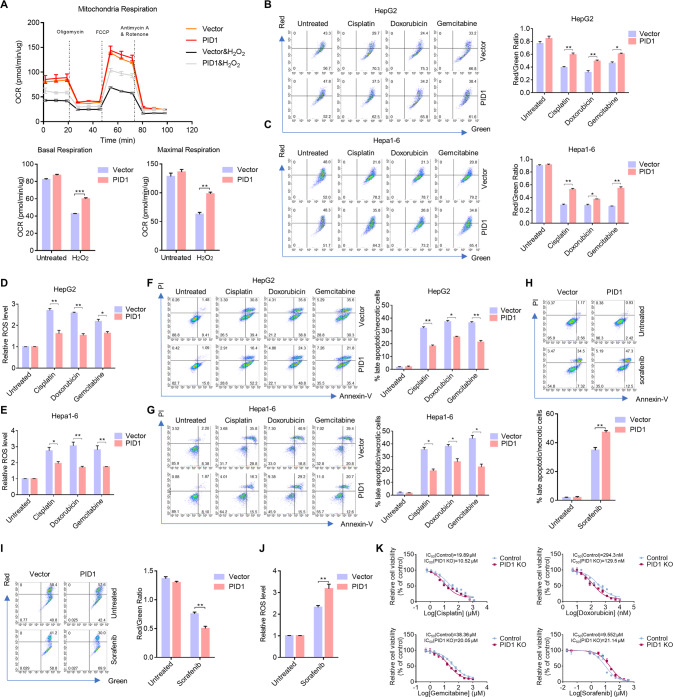
PID1 reduced efficacy of chemotherapeutic agents but facilitated Sorafenib-induced apoptosis. **A** Seahorse analysis was performed to evaluate the capacity of mitochondria respiration in HepG2 cells with or without PID1 overexpression upon H_2_O_2_ (200 μM) treatment for 12 h, basal respiration rate and maximum respiration rate were calculated. **B**–**C** Mitochondrial membrane potential in HepG2 cells and Hepa1–6 cells with or without PID1 overexpression upon treatment of three anticancer agents was detected by cytometry analysis and Red/Green ratio was calculated. **D**–**E** HepG2 cells and Hepa1–6 cells were treated with Cisplatin (20 μM), Doxorubicin (200 nM) and Gemcitabine (20 μM) for 24 h. Intracellular ROS in HepG2 cells and Hepa1–6 cells with or without PID1 overexpression upon treatment of three anticancer agents was detected by cytometry analysis and MFI was calculated. **F-G** Cell apoptosis in HepG2 cells and Hepa1–6 cells with or without PID1 overexpression upon treatment of three anticancer agents. **H** Cell apoptosis in HepG2 cells with or without PID1 overexpression upon Sorafenib (10 μM) treatment for 24 h was examined by cytometry analysis. **I** Mitochondrial membrane potential in HepG2 cells with or without PID1 overexpression upon Sorafenib (10 μM) treatment for 24 h was assessed by cytometry analysis and Red/Green ratio was calculated. **J** Intracellular ROS in HepG2 cells with or without PID1 overexpression upon Sorafenib (10 μM) treatment for 24 h was assessed by cytometry analysis and MFI was calculated. **K** Cell viability curves of Hep3B cells with or without PID1 deficiency treated with the indicated doses of anticancer agents for 24 h. Data are expressed as mean ± SD (*n* = 3). *p < 0.05; **p < 0.01; ***p < 0.001.

We performed PID1 overexpression in hepatoma cells with low PID1 expression levels (Fig. S1A). Interestingly, PID1 overexpression was found to inhibit oxidative stress-induced apoptosis and ROS production. (Fig. S1B, C). Moreover, PID1-expressing cells maintained higher MMP in response to H_2_O_2_ (Fig. S1D). Mitochondrial dysfunction is directly related to mitochondrial respiratory depression [22, 23]. We thus conducted Seahorse assay and found that HepG2 cells with PID1 overexpression sustained higher oxygen consumption rate upon H_2_O_2_ treatment (Fig. 2A). Mitochondria-dependent apoptosis is usually induced by various anticancer agents [24–27]. Conventional chemotherapeutic agents (Cisplatin, Doxorubicin and Gemcitabine) decreased MMP greatly, whereas PID1 could alleviate this effect (Fig. 2B, C). Correspondingly, PID1 inhibited ROS production (Fig. 2D, E) and apoptosis (Fig. 2F, G) induced by three distinct chemotherapeutic agents, suggesting that PID1 could protect against mitochondrial dysfunction induced by chemotherapeutic agents. Sorafenib is widely used in the clinical treatment of advanced HCC [5], hence we next determined whether PID1 also affected the pharmacological efficacy of Sorafenib. Surprisingly, PID1 overexpression not only aggravated Sorafenib-induced apoptosis, but also led to decline in MMP and increase in ROS production (Fig. 2H–J). We then performed PID1 knockout in PID1-amplified HCC cells: Hep3B (Fig. S1E). PID1 deficiency significantly enhanced efficacy of three chemotherapeutic agents but markedly decreased the sensitivity to Sorafenib (Fig. 2K). These results indicated that PID1 can affect apoptosis induced by different anticancer agents in a context-dependent manner.

### PID1 inhibited Doxorubicin-induced apoptosis via AKT/Raf-1/BAD axis

**Fig. 3:**
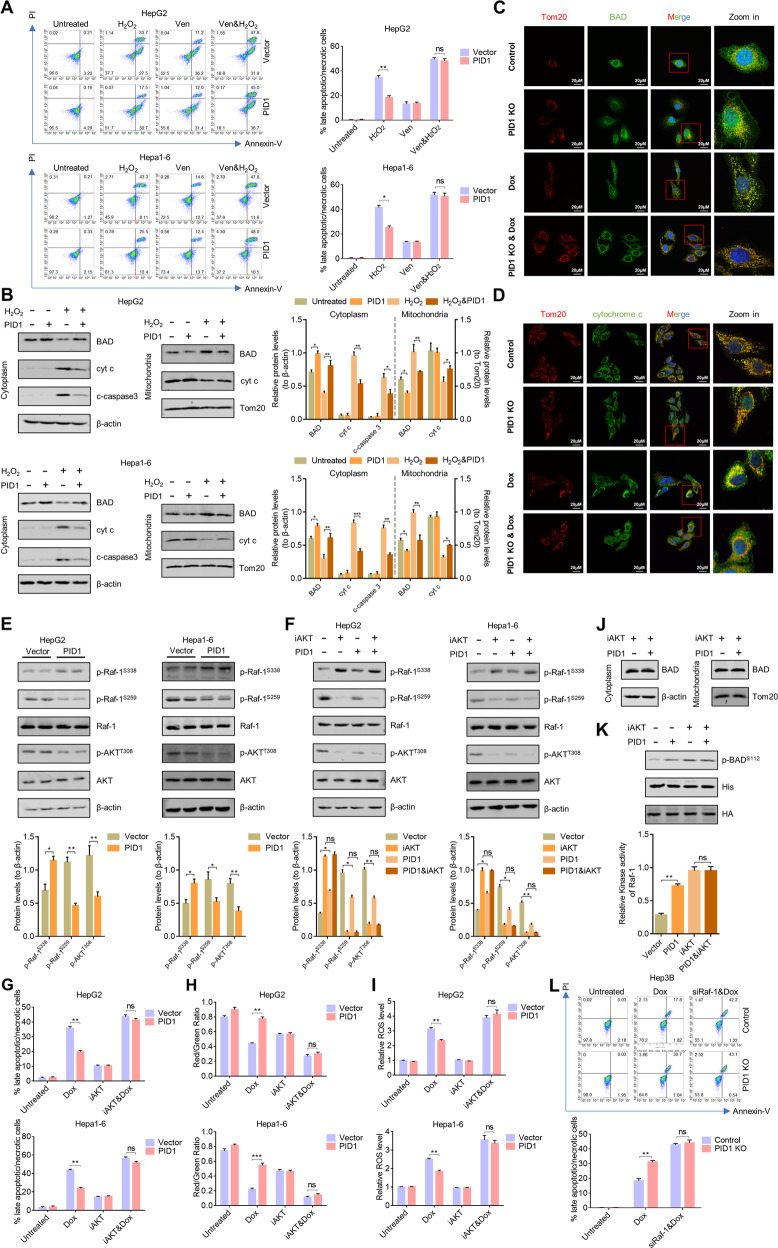
PID1 inhibited Doxorubicin-induced apoptosis via AKT/Raf-1/BAD axis. **A** Cell apoptosis in HepG2 cells and Hepa1–6 cells with or without PID1 overexpression upon H_2_O_2_ (200 μM) treatment for 24 h in the presence of Venetoclax (Ven) (10 μM). **B** BAD location, cytochrome c release and caspase-3 activation in response to H_2_O_2_ (200 μM) in HepG2 cells and Hepa1–6 cells with or without PID1 overexpression. Cytoplasmic fraction and mitochondrial fraction in cells were prepared. **C** Immunofluorescence staining of Tom20 and BAD in Hep3B cells upon indicated treatments. Merged images show the overlap of Tom20 (red), BAD (green) and nuclear staining by DAPI (blue). **D** Immunofluorescence staining of Tom20 and cytochrome c in Hep3B cells upon indicated treatments. Merged images show the overlap of Tom20 (red), cytochrome c (green) and nuclear staining by DAPI (blue). **E** Western blot analysis of Raf-1, p-Raf-1^S338^, p-Raf-1^S259^, AKT, p-AKT^T308^ and β-actin in HepG2 cells and Hepa1–6 cells with or without PID1 overexpression. **F** Western blot analysis of Raf-1, p-Raf-1^S338^, p-Raf-1^S259^, AKT, p-AKT^T308^ and β-actin in HepG2 cells and Hepa1–6 cells with or without PID1 overexpression in the presence of AKT inhibitor VIII (iAKT) (10 μM). **G** Cell apoptosis in HepG2 cells and Hepa1–6 cells with or without PID1 overexpression upon Doxorubicin (200 nM) treatment for 24 h in the presence of AKT inhibitor VIII (10 μM). **H** Mitochondrial membrane potential in HepG2 cells and Hepa1–6 cells with or without PID1 overexpression upon Doxorubicin (200 nM) treatment for 24 h in the presence of AKT inhibitor VIII (10 μM) was examined by cytometry analysis, and Red/Green ratio was calculated. **I** Intracellular ROS in HepG2 cells and Hepa1–6 cells with or without PID1 overexpression upon Doxorubicin (200 nM) treatment for 24 h in the presence of AKT inhibitor VIII (10 μM) was examined by cytometry analysis, and MFI was calculated. **J** HepG2 cells with or without PID1 overexpression were treated with AKT inhibitor VIII (10 μM) and mitochondria-located BAD levels were detected by western blot analysis. Cytoplasmic fraction and mitochondrial fraction in cells were prepared. **K** In vitro kinase assay was performed with bacterially purified His-BAD as substrate, and immunoprecipitated HA-Raf-1 in HepG2 cells with or without PID1 overexpression upon AKT inhibitor VIII treatment as kinase. Phosphorylation of His-BAD at Ser112 was detected by western blot analysis. **L** Cell apoptosis in Hep3B cells with or without PID1 knockout combined with Raf-1 knockdown upon Doxorubicin (200 nM) treatment for 24 h was examined by cytometry analysis. Data are expressed as mean ± SD (*n* = 3). *p < 0.05; **p < 0.01; ***p < 0.001, ns not significant.

We initially found that PID1 promoted phosphorylation of BAD at Ser112 significantly (Fig. S2A). To address the possible role of BAD in PID1-mediated resistance to apoptosis, Venetoclax (Ven), an inhibitor for Bcl2 as well as Bcl-xL, was utilized to block the targets of BAD, and PID1 failed to maintain its anti-apoptotic effect in the presence of Ven in HepG2 and Hepa1–6 cells (Fig. 3A). Correspondingly, PID1 decreased mitochondrial BAD levels in response to H_2_O_2_, combined with the reduction in release of cytochrome c and decreased cleaved-caspase3 levels (Fig. 3B), suggesting that PID1 inhibited apoptosis through suppressing BAD activity. Doxorubicin is widely used in conventional TACE and has been reported to induce BAD activation [28, 29]. We then performed immunofluorescence assay to investigate the effect of PID1 on Doxorubicin-induced apoptotic signals. By observing the overlap of red fluorescence (Tom20, translocase of mitochondrial membrane 20) and green fluorescence (BAD or cytochrome c), we found that PID1 knockout also facilitated Doxorubicin-induced translocation of BAD to mitochondria and led to increased release of cytochrome c from mitochondria in Hep3B cells (Figs. 3C, D, S2B, C).

Previous studies have indicated that PID1 could downregulate PI3K/AKT pathway [7, 30]. Importantly, p-AKT directly phosphorylates Raf-1 at Ser259 to inhibit Raf-1 activation, whereas activated Raf-1 has been found to phosphorylate BAD at Ser112 [16, 31]. We thus hypothesized that PID1 can possibly facilitate BAD phosphorylation through relieving AKT-mediated inhibition on Raf-1. In support of it, we observed that both p-AKT and p-Raf-1^S259^ levels were downregulated in PID1-expressing cells, combined with upregulation of p-Raf-1^Ser338^ levels (Fig. 3E). AKT inhibition has been reported to restrain IGF-1-induced phosphorylation of Raf-1 at Ser259 [32]. We herein observed that AKT inhibitor VIII (iAKT) also downregulated p-Raf-1^S259^ levels, and PID1 failed to further activate Raf-1 when AKT activation was inhibited (Fig. 3F). Correspondingly, iAKT aggravated Doxorubicin-induced apoptosis and PID1 failed to relieve it (Figs. 3G, S2D). In addition, PID1 did not affect Doxorubicin-induced ROS production and MMP decline when AKT was blocked (Figs. 3H, I, S2E), confirming that PID1 alleviated mitochondrial dysfunction in an AKT-dependent manner. Interestingly, PID1 also failed to decrease mitochondria-located BAD levels in the presence of iAKT (Fig. 3J). In vitro kinase assay showed that HA-Raf-1 in cells with PID1 overexpression demonstrated stronger activity to catalyze His-BAD phosphorylation at Ser112, whereas AKT inhibition abolished this effect (Fig. 3K). Moreover, PID1 deficiency failed to increase the sensitivity to Doxorubicin when Raf-1 was knocked down (Figs. 3L, S2F). These results indicated that PID1 could facilitate Raf-1-mediated BAD phosphorylation through relieving AKT-mediated inhibition on Raf-1, thus inhibiting Doxorubicin-induced apoptosis.

### PID1 inhibited continuous AKT activation via interacting with PDPK1

**Fig. 4:**
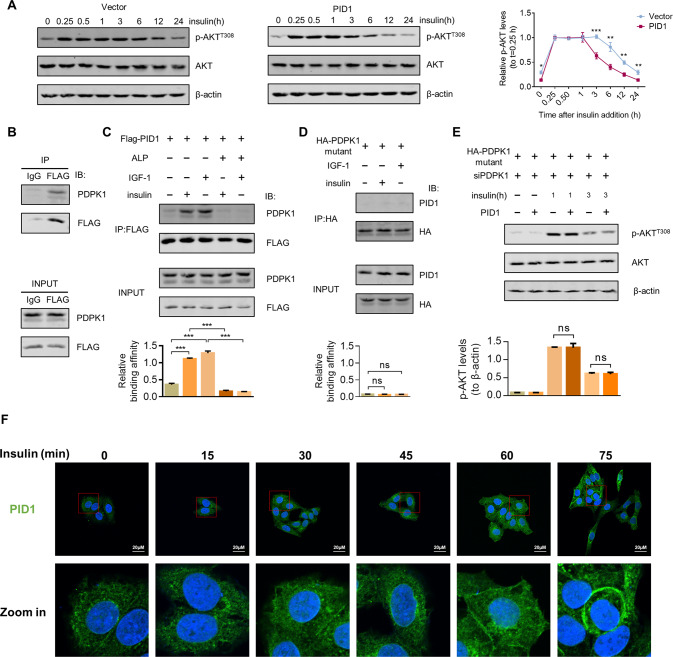
PID1 inhibited continuous AKT activation via interacting with PDPK1 but facilitated IGF-1-induced survival signal. **A** Time-course analysis of AKT phosphorylation upon insulin (10 nM) treatment in HepG2 cells with or without PID1 overexpression. p-AKT^T308^ band density was normalized to actin and then normalized to *t* = 0.25 h. **B** Interaction between endogenous PDPK1 and FLAG-tagged PID1 was confirmed by co-immunoprecipitation assay. **C** Interaction between endogenous PDPK1 and FLAG-tagged PID1 upon indicated treatment was examined by co-immunoprecipitation assay. **D** Interaction between endogenous PID1 and HA-tagged PDPK1 mutant (Y9, 373 and 376 F) upon indicated treatment was examined by co-immunoprecipitation assay. **E** Western blot analysis of AKT, p-AKT^T308^ and β-actin in HepG2 cells with indicated treatment was performed. **F** Immunofluorescence staining of PID1 in Hep3B cells upon treatment of insulin for indicated time. Merged images show the overlap of PID1 (green) and nuclear staining by DAPI (blue). Data are expressed as mean ± SD (*n* = 3). *p < 0.05; **p < 0.01, ***p < 0.001; ns not significant.

Although earlier studies have indicated that PID1 could inhibit PI3K/AKT pathway [7, 30], PID1-expressing cells retained the ability to respond to insulin (Fig. S3A). Time-course analysis revealed that PID1 inhibited AKT in a time-dependent manner but did not affect insulin-induced AKT activation within 1 h (Fig. 4A). Interestingly, previous reports have suggested that PDPK1 is constitutively active and can be further activated by tyrosine phosphorylation (Y9/373/376) following the activation of growth factor receptors, thereby contributing to continuous AKT activation [33–35]. Importantly, we found that endogenous PDPK1 was present in FLAG-tagged PID1 immunoprecipitates (Fig. 4B). Since PID1 contains a PTB domain, we postulated that PID1 may regulate AKT activation through interacting with PDPK1 at phosphorylated tyrosine sites. In support of it, growth factors enhanced the interaction between PDPK1 and FLAG-tagged PID1, whereas alkaline phosphatase (ALP) almost completely disrupted this interaction (Fig. 4C). We then constructed PDPK1 mutant (Y9, Y373 and Y376F), and found that growth factors failed to enhance the interaction between PID1 and HA-tagged PDPK1 mutant (Fig. 4D). Moreover, PID1 was unable to inhibit continuous AKT activation in cells with WT PDPK1 knockdown and ectopic expression of PDPK1 mutant (Figs. 4E, S2B). PDPK1 has been reported to translocate to the cell membrane following PI3K activation [36]. Importantly, PID1 did not translocate to membrane until 1 h after insulin treatment, indicating that PID1 interacted with PDPK1 and inhibited AKT activation with delayed dynamics (Fig. 4F). These results explained the reason behind the minimal effect of PID1 on insulin-induced AKT activation in the initial stages.

### PID1 promoted Sorafenib-induced apoptosis via blocking AKT activation

**Fig. 5:**
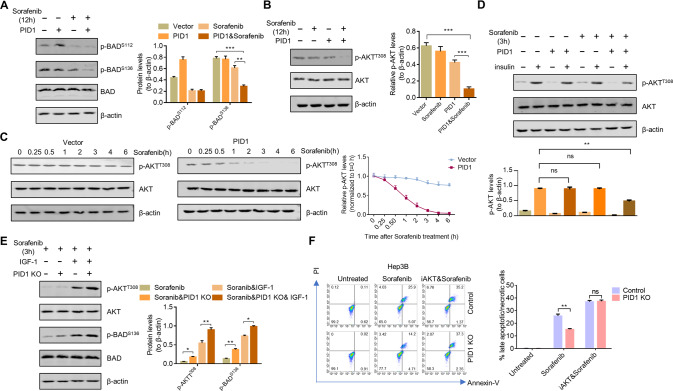
PID1 promoted Sorafenib-induced apoptosis via blocking AKT activation. **A** Western blot analysis of BAD, p-BAD^S112^, p-BAD^S136^ and β-actin in HepG2 cells with or without PID1 overexpression upon Sorafenib (10 μM) treatment for 12 h. **B** Western blot analysis of AKT, p-AKT^T308^ and β-actin in HepG2 cells with or without PID1 overexpression upon Sorafenib (10 μM) treatment for 12 h. **C** Time-course analysis of p-AKT^T308^ levels in HepG2 cells with or without PID1 overexpression upon Sorafenib (10 μM) treatment. **D** HepG2 cells with or without PID1 overexpression were pretreated with Sorafenib (10 μM) for 3 h and then treated with insulin (10 nM) for 0.25 h. Western blot analysis of AKT, p-AKT^T308^ and β-actin was performed. **E** Hep3B cells with or without PID1 deficiency were pretreated with Sorafenib (10 μM) for 3 h and then treated with IGF-1 (50 ng/mL) treatment for 0.25 h. Western blot analysis of AKT, p-AKT^T308^, BAD, p-BAD^S136^ and β-actin was performed. **F** Cell apoptosis in Hep3B with or without PID1 knockout upon Sorafenib (10 μM) treatment for 24 h in the presence of AKT inhibitor VIII (10 μM) was examined by cytometry analysis. Data are expressed as mean ± SD (*n* = 3). *p < 0.05; **p < 0.01; ***p < 0.001; ns not significant.

We subsequently investigated the potential role of PID1 in Sorafenib-induced apoptosis. Sorafenib completely blocked BAD phosphorylation at Ser112 regardless of PID1 levels, whereas PID1 significantly inhibited BAD phosphorylation at Ser136 upon Sorafenib treatment (Fig. 5A). Moreover, Sorafenib suppressed AKT more potently in PID1-expressing cells (Fig. 5B), suggesting that Sorafenib cooperated with PID1 to inhibit AKT-mediated survival signals. Time-course analysis revealed that PID1 accelerated Sorafenib-induced AKT inhibition (Fig.5C). Importantly, only PID1-expressing cells upon Sorafenib treatment showed blunted response to insulin, suggesting that it was PID1 in combination with Sorafenib that blocked AKT activation (Fig. 5D). In support of it, PID1 knockout in Hep3B cells enhanced IGF-1-mediated BAD phosphorylation when treated with Sorafenib (Fig. 5E). Moreover, PID1 deficiency attenuated Sorafenib-induced apoptosis, and AKT inhibition abolished this effect (Fig. 5F). Hence, our results indicated that PID1 could act in conjunction with Sorafenib to block AKT activation, thereby promoting Sorafenib-induced apoptosis.

### PID1 altered the rhythmicity of pharmacological activity of Sorafenib on Raf-1 and AKT

**Fig. 6:**
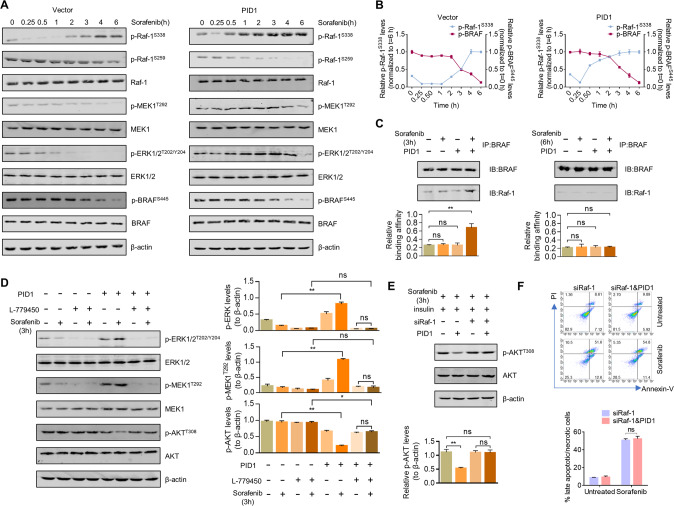
PID1 altered the rhythmicity of pharmacological activity of Sorafenib on Raf-1 and AKT. **A** Time-course analysis of p-Raf-1^S338^, p-Raf-1^S259^, p-MEK1^T292^, p-ERK1/2^T202/Y204^, BRAF and p-BRAF^S445^ in HepG2 cells with or without PID1 overexpression upon Sorafenib (10 μM) treatment. **B** Dynamics of Raf-1 activation and BRAF inactivation in HepG2 cells with or without PID1 overexpression upon Sorafenib (10 μM) treatment. p-Raf-1^S338^ band density was normalized to actin and then normalized to *t* = 6 h. p-BRAF^S445^ band density was normalized to actin and then normalized to *t* = 0. **C** Endogenous interaction between Raf-1 and BRAF in HepG2 cells with or without PID1 overexpression upon Sorafenib (10 μM) treatment for 3 h or 6 h was examined by co-immunoprecipitation assay. **D** Western blot analysis of ERK1/2, p-ERK1/2^T202/Y204^, MEK1, p-MEK1^T292^, AKT, p-AKT^T308^ and β-actin in HepG2 cells with or without PID1 overexpression upon Sorafenib (10 μM) treatment for 3 h in the presence of L-779450 (20 μM). **E** HepG2 cells with or without PID1 overexpression upon Raf-1 knockdown were pretreated with Sorafenib (10 μM) for 3 h and then treated with insulin for 0.25 h. Western blot analysis of AKT, p-AKT^T308^ and β-actin was performed. **F** Cell apoptosis in HepG2 with or without PID1 overexpression combined with Raf-1 knockdown upon Sorafenib (10 μM) treatment for 24 h was examined by cytometry analysis. Data are expressed as mean ± SD (*n* = 3). *p < 0.05; **p < 0.01; ns not significant.

Notably, Sorafenib, known for multi-kinase inhibition including Raf-1 inhibition [37], has been found to paradoxically activate Raf-1 significantly (Fig. S4A). Time-course analysis revealed that Sorafenib inhibited Raf-1 significantly within few minutes and then gradually activated Raf-1 (Fig. 6A). Of note, PID1 accelerated Sorafenib-induced Raf-1 activation in an AKT-dependent manner (Fig. S4B). Moreover, ERK hyperactivation was induced transiently in PID1-expressing cells (Fig. 6A). Previous studies indicated that ERK hyperactivation led to phosphorylation of MEK1 at Thr292, thus inhibiting AKT and ERK significantly [38]. We also found that phosphorylation of MEK1 at Thr292 was induced with similar dynamics of ERK in PID1-expressing cells (Figs. 6A, S4C), accounting for PID1-mediated AKT blockade in response to Sorafenib.

Prior studies have indicated that activated Raf-1 could form heterodimer with activated BRAF, thereby activating ERK significantly [39–41]. In support of it, Raf-1 selective inhibitors (ZM336372 and GW5074), activate not only Raf-1 but also ERK, which differed from action of Sorafenib and could be abrogated by BRAF selective inhibitor L-779450 (Fig. S4D, E).

We next investigated the involvement of Raf-1/BRAF heterodimerization in PID1-mediated AKT blockade in response to Sorafenib. Sorafenib inhibited BRAF gradually, which differed from its pharmacological activity on Raf-1 (Fig. 6B). While, PID1 accelerated Sorafenib-induced Raf-1 activation, causing simultaneous activation of Raf-1/BRAF and heterodimerization (Fig. 6B). Correspondingly, endogenous Raf-1 was only detected in BRAF immunoprecipitates in PID1-expressing cells treated with Sorafenib for 3 h, but not for 6 h (Fig. 6C). BRAF inhibition or Raf-1 depletion removed Sorafenib-induced barrier in AKT activation in PID1-expressing cells (Fig. 6D, E). Besides, PID1 failed to facilitate Sorafenib-induced apoptosis after Raf-1 depletion (Fig. 6F). Hence, these results indicated that PID1 could accelerate Sorafenib-induced Raf-1 activation and facilitate the formation of Raf-1/BRAF heterodimerization, thereby blocking AKT activation via Raf-1-dependent pathway and leading to increased sensitivity of hepatoma cells to Sorafenib.

### PID1 reduced efficacy of Doxorubicin but facilitated Sorafenib-induced apoptosis in vivo

**Fig. 7:**
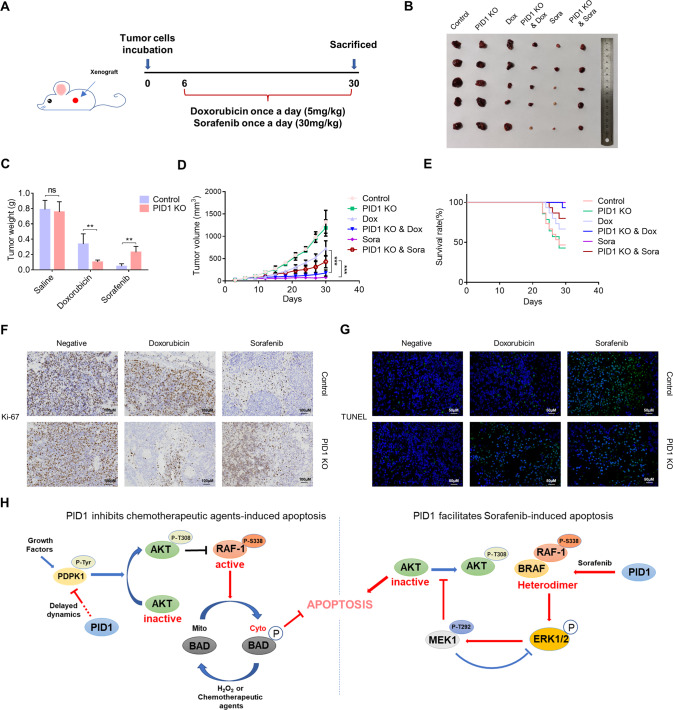
PID1 reduced efficacy of Doxorubicin but facilitated Sorafenib-induced apoptosis in vivo. **A** The diagram of therapy design. Hep3B cells with or without PID1 knockout were subcutaneously inoculated in the right flanks of mice. Mice were randomly divided into six groups when they first developed a mass at day 6 (*n* = 15). Doxorubicin and Sorafenib were given intraperitoneally with a daily dosage of 5 mg/kg for Doxorubicin and 30 mg/kg for Sorafenib until the end point at day 30. **B** Photographs illustrating tumors in xenografts of Hep3B cells with or without knockout upon indicated treatments (*n* = 5). **C** Weight of xenograft tumors. **D** Growth curves of xenograft tumors upon indicated treatments. **E** Survival curve showed that PID1 deficiency increased survival rate upon Doxorubicin treatment but declined efficacy of Sorafenib. **F** Ki-67 staining of tumor slices. **G** TUNEL staining of tumor slices. **H** Model for the mechanism by which PID1 inhibits conventional chemotherapeutic agents-induced apoptosis but promotes Sorafenib-induced apoptosis.

To determine whether the above findings could be translated in vivo, we established mouse HCC model by subcutaneously injecting Hep3B cells with or without PID1 knockout into nude mice, and schematic representation of the therapy design has been shown in Fig. 7A. It was observed that PID1 deficiency evidently decreased tumor growth in Doxorubicin-treated nude mice but restrained the anti-tumor potential of Sorafenib (Fig. 7B). The inhibitory efficiency of Doxorubicin on the weight and volume of tumors were strengthened by PID1 knockout, whereas opposite results were obtained with Sorafenib (Fig. 7C, D). Mice bearing tumors with PID1 deficiency showed a significant survival advantage upon Doxorubicin treatment but demonstrated poor prognosis when treated with Sorafenib (Fig. 7E). Moreover, PID1 deficiency resulted in significantly reduced expression of proliferation marker Ki-67 and increased apoptosis as evident by TUNEL staining in response to Doxorubicin but showed opposite effects upon Sorafenib treatment (Fig. 7F, G). These results established PID1 as a novel biomarker of resistance to Doxorubicin but sensitive to Sorafenib in preclinical HCC model.

## Discussion

It has established that Sorafenib resistance can lead to ineffective treatment of advanced HCC and limited response to conventional chemotherapeutic agents can restrict efficiency of TACE in intermediate HCC [3, 4, 28]. While DNA or enzymes required for DNA synthesis are major targets of conventional chemotherapeutic agents, Sorafenib inhibits various oncogenic kinases such as Raf-1, BRAF, VEGFR and PDGFR-β, explaining for different anticancer spectrum of these two drug classes [1, 2, 5]. Despite discrepancy in the pharmacological activity, anticancer agents usually induce apoptosis via mitochondria-dependent pathway [42]. Mitochondria homeostasis is primarily regulated by Bcl2 family and BAD is one of pro-apoptotic Bcl2 family members, normally present in inactive state and sequestered in the cytoplasm by YWHAZ [43]. The function of BAD could be effectively inhibited by Raf-1-mediated phosphorylation at Ser112 or AKT-mediated phosphorylation at Ser136 [31, 44]. Once dephosphorylated by the pro-apoptotic stimuli such as growth factor deprivation or chemotherapeutic agents, BAD can target mitochondria and promote cell death [45, 46]. A number of prior studies have suggested that inhibition of BAD phosphorylation sensitizes cancer cells to anticancer agents [47–50]. Importantly, Sorafenib can directly inhibit kinase activity of Raf-1 and affect AKT activation indirectly via blocking VEGFR or PDGFR-β [2]. Thus, Sorafenib directly involves in BAD regulation, which differs significantly from the conventional chemotherapeutic agents. Hence, increased understanding of BAD-mediated apoptosis might be helpful for guiding clinical treatment.

PID1 was initially found in adipose tissues and has been implicated in the regulation of various biological processes [8]. We herein reveal that PID1 exhibited broad-spectrum of anti-apoptotic activity for conventional chemotherapeutic agents but facilitated Sorafenib-induced apoptosis. This paradoxical phenomenon originates from complex dynamics of PID1-mediated AKT inhibition. PID1 inhibits continuous AKT activation rather than blocking growth factors induced AKT activation. Although PID1 was reported to induce insulin resistance in the transgenic mice, hepatoma cells with PID1 overexpression still retained the capacity to respond to insulin in our study [9, 51]. A recent study has suggested that adipose tissues rather than adipocytes in obesity subjects were unable to respond to insulin, which partly explained for differential roles of PID1 [52]. Interestingly, PID1 can interact with PDPK1 to inhibit AKT activation and this process depends on the phosphorylation of PDPK1 at tyrosine site (Y9/373/376). Since PDPK1 is recruited to cytomembrane to activate AKT once PI3K is activated and then gets phosphorylated at the tyrosine sites, growth factor receptors-mediated activation of PI3K/AKT pathway follows a specific order, that is AKT activation occurs first, PID1 thereafter interacts with PDPK1 at the phosphorylated tyrosine sites and inhibits continuous AKT activation later.

Importantly, PID1-mediated AKT inhibition can facilitate Raf-1 activation. Raf-1 is regulated by several phosphorylation sites [15]. In brief, phosphorylation of Raf-1 at Ser338 residue has been found to be indispensable for Raf-1 activation, and phosphorylation at Ser259 results in Raf-1 inactivation [15]. Intriguingly, p-AKT can directly phosphorylate Raf-1 at Ser259 and inhibit Raf-1, thus emphasizing the involvement of AKT in Raf-1 activation [16, 53]. It is worth noting that the function of BAD is mainly regulated by AKT and Raf-1, and its phosphorylation is tightly related to chemotherapy failure. We found that PID1 facilitated Raf-1-mediated phosphorylation of BAD on Ser112 by restricting continuous AKT activation, thereby inhibiting apoptosis and leading to the reduced efficacy of conventional chemotherapeutic agents.

However, PID1 facilitated Sorafenib-induced apoptosis via AKT/Raf-1-dependnet pathway. Raf-1 is the major target of Sorafenib and AKT activation is directly related to Sorafenib resistance [37, 54–56]. Sorafenib is reported to abnormally activate Raf-1 in negative feedback manner [57–59]. Similarly, findings of our study confirmed that Sorafenib inhibited Raf-1 rapidly within few minutes, and then gradually activated Raf-1. Importantly, PID1 accelerated Sorafenib-induced Raf-1 activation and caused a transient excessive ERK activation followed by AKT inhibition, indicating the existence of underlying interactions among PID1, Raf-1, ERK and AKT. Raf-1 is known to activate ERK, but prior studies have indicated that Raf-1/BRAF heterodimer can activate ERK more potently [39–41]. Importantly, BRAF is always maintained in an active state, suggesting that the formation of Raf-1/BRAF heterodimer depends on Raf-1 activation [60]. Therefore, ZM336372 and GW5074, two Raf-1 selective inhibitors, paradoxically activated Raf-1 and exhibited powerful effect on ERK activation but failed to stimulate ERK in the presence of BRAF inhibitor L-779450. However, Sorafenib treatment led to slow but persistent BRAF inhibition, explaining the powerful inhibitory effect of Sorafenib on ERK. Overall, our results revealed that PID1 can accelerate Sorafenib-induced Raf-1 activation and enable simultaneous activation of Raf-1 and BRAF, thereby facilitating Raf-1/BRAF heterodimerization and subsequent ERK activation. It has been reported that excessive ERK activation can inhibit both ERK and AKT by phosphorylating MEK1 at Thr292 [61, 62]. Moreover, MEK1 knock-out (not MEK2) can lead to activation of MAPK/ERK and PI3K/AKT pathway, supporting that ERK-mediated feedback mechanism involves in suppression of AKT activity [38]. It is worth noting that only PID1-expressing cells with Sorafenib treatment showed blunted response to insulin, further confirming that it is PID1 in combination with Sorafenib that blocks AKT activation. We also noted that PID1 synergized with Sorafenib to transiently phosphorylate MEK1 at Thr292 significantly, and both BRAF inhibition and Raf-1 depletion disrupted the Sorafenib-induced barrier in AKT activation. Therefore, PID1 can alter the rhythmicity of pharmacological process of Sorafenib on different survival-related kinases, thus blocking AKT activation in a Raf-1-dependent manner and resulting in increased efficacy of Sorafenib.

Although anticancer agents can always lead to mitochondria-dependent apoptosis, different agents exhibit diverse mechanisms of actions [3, 6, 42]. Conventional chemotherapeutic agents block processes such as DNA replication and transcription, leading to the cell cycle arrest and thus apoptosis [1, 2, 4]. Hence, PID1 exhibits a broad-spectrum anti-apoptotic activity for the conventional chemotherapeutic agents by enhancing Raf-1-mediated survival signals. On the contrary, Sorafenib, a multi-kinase inhibitor can directly affect kinases related to survival and PID1 can act in conjunction with it to block AKT-mediated survival signals through Raf-1-dependent pathway, thus leading to increased apoptosis. Hence, PID1 is just like the same “knife” that cuts both the bread and fingers, and its role in apoptosis depends upon the drug applied for the treatment.

Overall, we describe a PID1-mediated, dynamics-driven mechanism that can regulate apoptosis induced by diverse anticancer agents in hepatoma cells. These results identify PID1 as a signature of resistance to chemotherapeutic agents such as Doxorubicin but also an underlying biomarker of sensitivity to Sorafenib, which may be helpful for guiding clinical treatment.

## Materials and Methods

### Cell culture

The human hepatoma cell lines, HepG2, Hep3B and SK-Hep-1 were purchased from Procell (Wuhan, China). The mice hepatoma cell lines, H22 and Hepa1–6 were purchased from the China Center for Type Culture Collection (Wuhan, China). All the cell lines were maintained in Dulbecco’s Modified Eagle Medium (DMEM)-high glucose supplemented with 12% FBS in the presence of 1% penicillin/streptomycin at 37 °C under the atmosphere of 95% air and 5% CO_2_. The cell lines were identified by STR profiling and relative information is available in supplementary files.

### Cell transfection and infection

The human expression plasmids (FLAG-tagged PID1, HA-tagged Raf-1, His-tagged BAD and HA-tagged PDPK1 mutant) and mice PID1 expression plasmid were obtained from Gene Create (Wuhan, China). The cancer cell lines were transiently transfected with empty vector or PID1 expression plasmid using Neofect^TM^ (Neofect Biotech, Beijing, China). The transfected cells were assessed for PID1 expression by western blot analysis. Specific siRNA targeting Raf-1 (Raf-1 si#1: 5’-AAACUCAUCGCUCAUCCUUCG-3’; Raf-1 si#2: 5’-AUCUGUAGCACUAGCGUCUUC-3’; Raf-1 si#3: 5’-UUUGCCCAAGUUUCGAUCCCA-3’) were obtained from Gene Create. The cells were transfected with Raf-1 siRNA using Hieff Trans (YEASON, Shanghai, China). The silencing efficacy of the respective siRNAs was confirmed by western blot analysis. For lentiviral infection, Hep3B cells were incubated with lentivirus-CRISPR/Cas9-puro-PID1 KO construct (sgRNA1#1: GGCAGTCCATCTGGTAGGAC; sgRN1#2: TCATCTCGACCACAAAGGGG; sgRNA#3: AGATGTTGGGGCTCACGTTG) at MIO of 20 for 24 h, and then treated with 2 μg/mL puromycin for 72 h.

### Cell treatment

The cultured cells transfected with PID1 expression plasmid for 24 h were then harvested for the subsequent experiments. The specific inhibitors targeting AKT (AKT inhibitor VIII, HY-10355), Bcl2 and Bcl-xL (Venetoclax, HY-15531), Raf-1 (ZM336372 HY-13343; GW5074 HY-10542) as well as BRAF (L-779450, HY-12787) were purchased from MCE (Shanghai, China). The selected inhibitor was added to medium 4 h before H_2_O_2_ or Sorafenib treatment and subsequent experiments were then performed.

### Cell death assay

The cultured cells were collected by combing the floating cells in the medium and adherent cells were treated with 0.25% trypsin, then washed three times with cold PBS. The cells were subsequently incubated with Annexin V-FITC and Propidium Iodide for 15 min at 37 °C in the dark. Apoptosis was analyzed by cytometric analysis.

### Mitochondrial membrane potential assay

The cultured cells were collected by combing the floating cells in medium and adherent cells were treated with 0.25% trypsin, then washed three times with cold PBS. The cells were subsequently incubated with JC-1 probes for 30 min at 37 °C in the dark and analyzed by flow cytometric analysis and Red/Green ratio was calculated.

### Intracellular ROS assay

The cultured cells were collected by combing floating cells in the medium and adherent cells were treated with 0.25% trypsin, then washed three times with cold PBS. The cells were subsequently stained with H_2_DCFDA probe for 30 min at 37 °C in the dark, washed three times with PBS and resuspended in PBS for analysis of ROS using cytometry analysis. Thereafter, mean fluorescence intensity (MFI) was calculated.

### Western blot analysis

The total protein was extracted from cells using RIPA lysis buffer in the presence of protease inhibitor cocktail and phosphatase inhibitor cocktail. The protein lysates were thereafter obtained by centrifugation at 12000 g for 10 min at 4 °C. Equal amounts of protein were loaded alongside a pre-stained protein ladder. The proteins were separated and then transferred to the nitrocellulose membrane. The membranes were blocked with Tris-buffered saline with Tween 20 in the presence of 5% skimmed milk at 25 °C for 1 h. The membranes were incubated with primary antibodies at 4 °C for 16 h. After washing thrice with TBST, the membranes were incubated with secondary antibodies conjugated with DyLight^TM^ 800 4XPEG fluorescent dye (Cell Signaling Technology, Danvers, MA, USA). Finally, near infrared detection system was used to detect the signal. Antibodies used to determine the protein expression were the following: PID1 (#27951; Signalway Antibody, Greenbelt, Maryland, USA), β-actin (#66009-1-lg; Proteintech, USA), Bcl-xL (#2764; Cell Signaling Technology, USA), Bcl2 (#12789-1-AP; Proteintech), BAD (#sc-8044 Santa Cruz Technology, Texas, USA), p-BAD^S112^ (#5284; Cell Signaling Technology), p-BAD^S136^ (#4366; Cell Signaling Technology), cytochrome c (#sc-13156; Santa Cruz Technology), cleaved caspase-3 (#9664; Cell Signaling Technology), Tom20 (#42406; Cell Signaling Technology), Raf-1 (#9422; Cell Signaling Technology), p-Raf-1^S259^ (#9421; Cell Signaling Technology), p-Raf-1^S338^ (#9427; Cell Signaling Technology), AKT (#4691; Cell Signaling Technology), p-AKT^T308^ (#13038; Cell Signaling Technology), HA-tag (#sc-7392; Santa Cruz Technology), His-tag (#sc-8036; Santa Cruz Technology), HA-tag (#14793; Cell Signaling Technology), His-tag (#12698; Cell Signal Technology), FLAG-tag (#3724; Cell Signaling Technology), FLAG-tag (#8146; Cell Signaling Technology), ERK1/2 (#4695; Cell Signaling Technology), p-ERK1/2^T202/Y204^ (#4370; Cell Signaling Technology), MEK1 (#9122; Cell Signaling Technology), p-MEK1^T292^ (#07-852; Upstate, Darmstadt, Germany), BRAF (#sc-5284; Santa Cruz Technology), p-BRAF^S445^ (#2696; Cell Signaling Technology).

### Mitochondrial and cytoplasmic isolation

Mitochondrial and cytoplasmic fractions from different lines were isolated by using Mitochondria isolation kit (Beyotime, Shanghai, China). Briefly, the collected cells were incubated with mitochondria isolation reagent for 15 min and homogenized using micro-homogenizer. The cytoplasmic fraction (pellet) was separated from cell homogenate by centrifugation at 1000 g for 10 min at 4 °C. The supernatant was further centrifugated at 3500 g for 15 min at 4 °C, and the mitochondrial fraction (pellet) was collected. The lysis of mitochondrial and cytoplasmic fractions was performed by addition RIPA lysis buffer containing protease inhibitor cocktail as well as phosphatase inhibitor cocktail, then protein lysates were obtained by centrifugation at 12000 g for 10 min at 4 °C.

### CCK-8 assay

The cultured cells with or without PID1 overexpression were seeded in 96-well plates at a density of 10000 cells per well. After an overnight incubation, the cells were treated with indicated doses of H_2_O_2_ or Sorafenib for 24 h, and then 10 μL CCK-8 was added to each well followed by 4 h incubation. The absorbance was detected at 450 nm, and IC50 was then calculated according to cell viability curves.

### Co-immunoprecipitation

The cultured cells were lysed in RIPA lysis buffer containing protease inhibitor cocktail and phosphatase inhibitor cocktail. The proteins were then immunoprecipitated from the cell lysates by incubating them with indicated antibodies for 2 h at 4 °C followed by incubation with Protein A/G agarose beads (sc-2003; Santa Cruz Technology) for 12 h at 4 °C. Subsequently, the immunoprecipitates were collected by centrifugation at 1000 g for 5 min and washed three times by using lysis buffer. Western blot analysis was then performed as described above.

### Immunofluorescence

The cells were seeded into 24-well dishes and pretreated with Doxorubicin for 24 h. After fixing in 4% paraformaldehyde for 10 min, the cells were permeabilized in 0.1% Triton-100 for 2 min and blocked with 5% goat serum, and were incubated with Tom20, cytochrome c and BAD antibodies overnight at 4 °C, followed by incubated with Alexa Fluor594-conjugate anti-rabbit (#8889; Cell Signal Technology) and Alexa Fluor488-conjugate anti-mouse (#4408; Cell Signal Technology) antibodies for detection. After staining with DAPI (Sigma, St Louis, MO, USA), the samples were photographed by laser confocal scanning microscope (Nikon, Tokyo, Japan).

### Kinase assay

HA-fusion Raf-1 protein protein was immunoprecipitated from HepG2 cells transfected with HA-tagged Raf-1 plasmids upon exposure to different treatment conditions. 1 μg of bacterially purified His-fusion BAD protein was incubated with immunoprecipitated HA-fusion Raf-1 protein in the kinase assay buffer (40 mM Tris-HCl pH 7.4, 1 mM dithiothreitol, 1 mM MnCl2, 1 mM EGTA) in the presence of 100 μM ATP at 30 °C for 20 min. The reaction was initiated by the addition of His-fusion BAD protein and subsequently stopped by the addition of SDS loading buffer. The phosphorylation of His-fusion BAD at Ser112 was then detected by western blot analysis as described above.

### Seahorse assay

Seahorse XFe24 Flux Assay Kit was used to measure the oxygen consumption rate (OCR). Briefly, HepG2 cells with or without PID1 overexpression were seeded in utility plate followed by incubation for 24 h and then treated with H_2_O_2_ for 12 h. Subsequently, the cells were washed with Seahorse buffer. Oligomycin, FCCP and Antimycin A/Rotenone were automatically injected into each well and OCR analysis was performed by Seahorse analysis system. The total protein levels of cells in each well were assessed by using BCA kit. The OCR values were then calculated and normalized to the total protein levels.

### Immunohistochemistry and TUNEL assay

The tumor slices were fixed in 4% paraformaldehyde, followed by dehydration and paraffin embedding. The sodium citrate buffer (pH = 6.0) was used for antigen retrieval and 0.3% NaHB_4_ was employed for quenching endogenous peroxidase activity. The sections were then blocked and incubated with primary antibodies at 4 °C overnight, followed by incubation with the secondary antibodies. The Vectastain Elite ABC Kit (Vector Laboratories, USA) and 3, 30-DAB were then used to detect the signals. TUNEL assay was performed following the protocol of In Situ Cell Death Detection Kit, Fluorescein (Roche, Burlington, MA, USA). The sections were deparaffinized and pretreated with Proteinase K for 25 min, then incubated in TUNEL reaction mixture for 1 h at 37 °C. The samples were photographed by laser confocal scanning microscope.

### Xenograft tumor mouse model

Five-week-old BALB/c nude mice (male) were subcutaneously injected with 3 × 10^6^ Hep3B cells with or without PID1 knockout. The tumor size was monitored every three days by measuring the length and width of the tumor. The tumor volume was calculated according to the formula V(mm^3^) = 1/2 × Length × Width^2^. After 6 days, the mice were treated with Doxorubicin (5 mg/kg) or Sorafenib (30 mg/kg) daily until being sacrificed (The doses were determined based on the preliminary study). Then 30 days after cell injection, the mice were anesthetized with 1.25% pentobarbital, and the tumors were resected and immersed in 4% paraformaldehyde. The animal study was approved by the Institutional Animal Care and Use Committee of Tongji Medical College, Huazhong University of Science and Technology ([2022] IACUC Number:2958). All animal experiments were conducted by following the ARRIVE guidelines. Randomization and single blinding were done in the entire experimental process. No statistical method was used for sample size estimation.

### Statistical analysis

The sample size was chosen according to references which conducted similar experiments and obtained significant results, or our preliminary experiments. All experiments were repeated at least three times and experimental data has been presented as mean ± SD. The comparisons between two groups were analyzed by Student’s t-tests (unpaired, two-tailed). In addition, Experiments with more than three groups were analyzed by one-way analysis of variance (ANOVA) plus Tukey’s post-hoc test. Statistical analysis was performed by using GraphPad Prism 6 software (San Diego, CA, USA). The differences were identified as significant at *p* < 0.05. *p < 0.05; **p < 0.01; ***p < 0.001.

## Supplementary information

Figure supplementary materials

STR sites information

Original Data File

## Data Availability

The data that support the findings of this study are available from the corresponding author upon reasonable request.
