# Supplementary material for: Dual role of PID1 in regulating apoptosis induced by distinct anticancer-agents through AKT/Raf-1-dependent pathway in hepatocellular carcinoma
Source: Cell Death Discov. 2023 Apr 28;9:139. doi: 10.1038/s41420-023-01405-1 (PMC10147665; doi:10.1038/s41420-023-01405-1)
Supplement: Supplementary file 1 — Figure supplementary materials [file 41420_2023_1405_MOESM1_ESM.docx]

**Supplementary Figures and Figure Legends:**


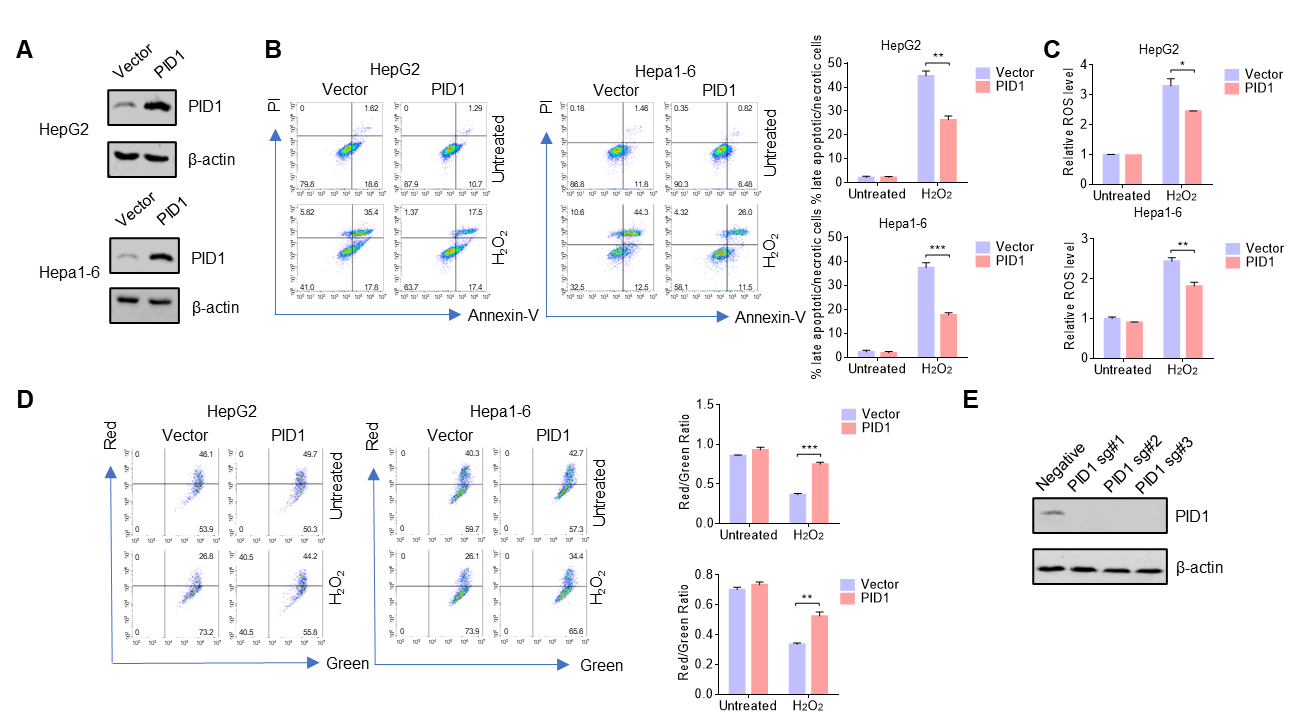


**Fig. S1** **A** Western blot analysis of PID1 and β-actin in HepG2 cells and Hepa1-6 cells transfected with human or mice PID1 cDNA. **B** Comparation of apoptosis induction in HepG2 cells and Hepa1-6 cells with or without PID1 overexpression upon H_2_O_2_ (200 μM) treatment for 24 h. **C** Intracellular ROS in HepG2 cells and Hepa1-6 cells with or without PID1 overexpression upon H_2_O_2_ (200 μM) treatment for 24 h was detected by cytometry analysis and MFI was calculated. **D** Mitochondrial membrane potential in HepG2 cells and Hepa1-6 cells with or without PID1 overexpression upon H_2_O_2_ (200 μM) treatment for 24 h was detected by cytometry analysis and Red/Green ratio was calculated. E, Western blot analysis of PID1 and β-actin in Hep3B cells with PID1 knockout.


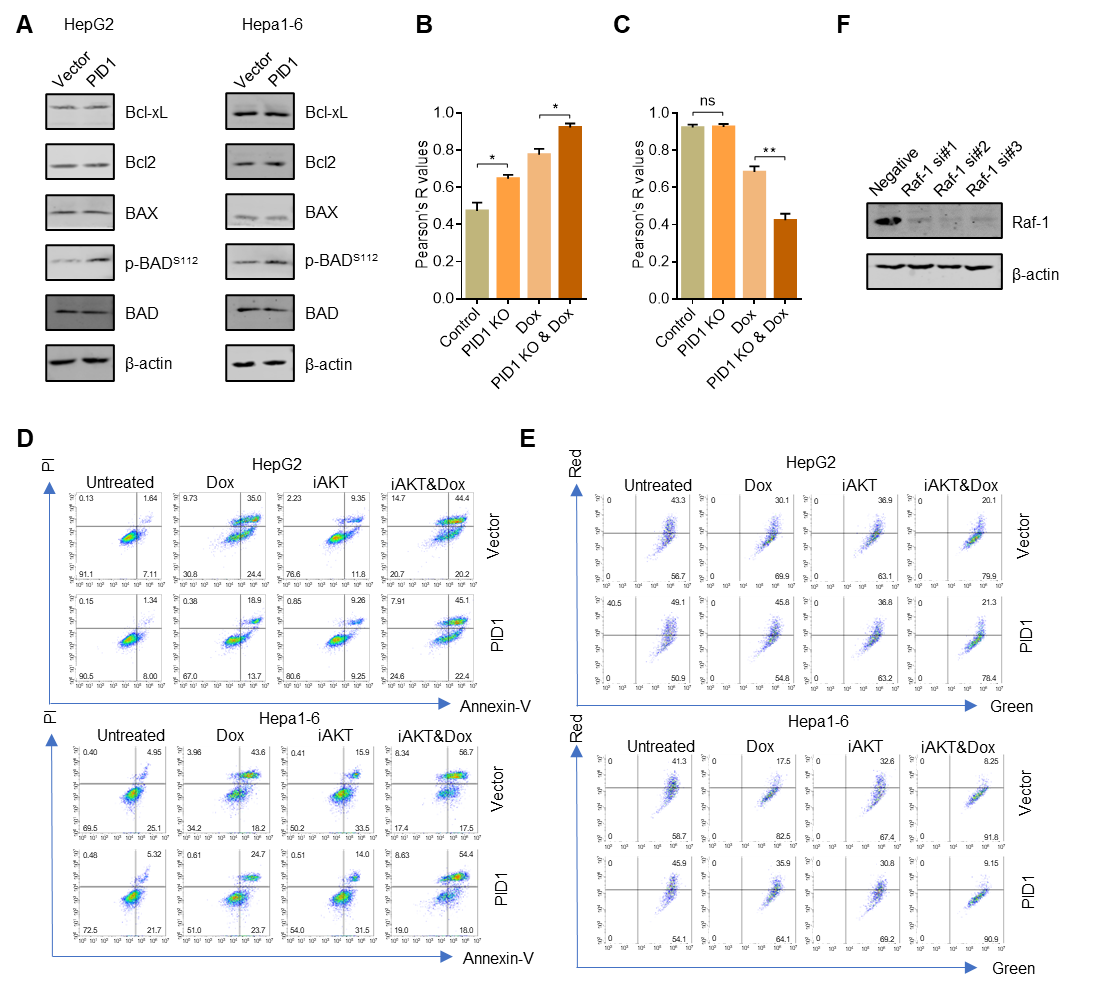


**Fig. S2 A** Western blot analysis of Bcl-xL, Bcl2, BAX, BAD, p-BAD^S112^ and β-actin in HepG2 cells and Hepa1-6 cells with or without PID1 overexpression. **B** Immunofluorescence staining of Tom20 and BAD in Hep3B cells upon indicated treatments. Pearson’s R values were calculated via Image J to represent the colocalization of BAD and mitochondria. **C** Immunofluorescence staining of Tom20 and cytochrome c in Hep3B cells upon indicated treatments. Pearson’s R values were calculated via Image J to represent the colocalization of cytochrome c and mitochondria. **D** Cell apoptosis in HepG2 cells and Hepa1-6 cells with or without PID1 overexpression upon H_2_O_2_ (200 μM) treatment for 24 h in the presence of AKT inhibitor VIII (10 μM). **E** Mitochondrial membrane potential in HepG2 cells and Hepa1-6 cells with or without PID1 overexpression upon H_2_O_2_ (200 μM) treatment for 24 h in the presence of AKT inhibitor VIII (10 μM) was examined by cytometry analysis. **F** Western blot analysis of Raf-1 and β-actin in Hep3B cells with Raf-1 knockdown. Data are expressed as mean ± SD (n=3). *, *p*<0.05; **, *p*<0.01; ***, *p*<0.001, ns, not significant.


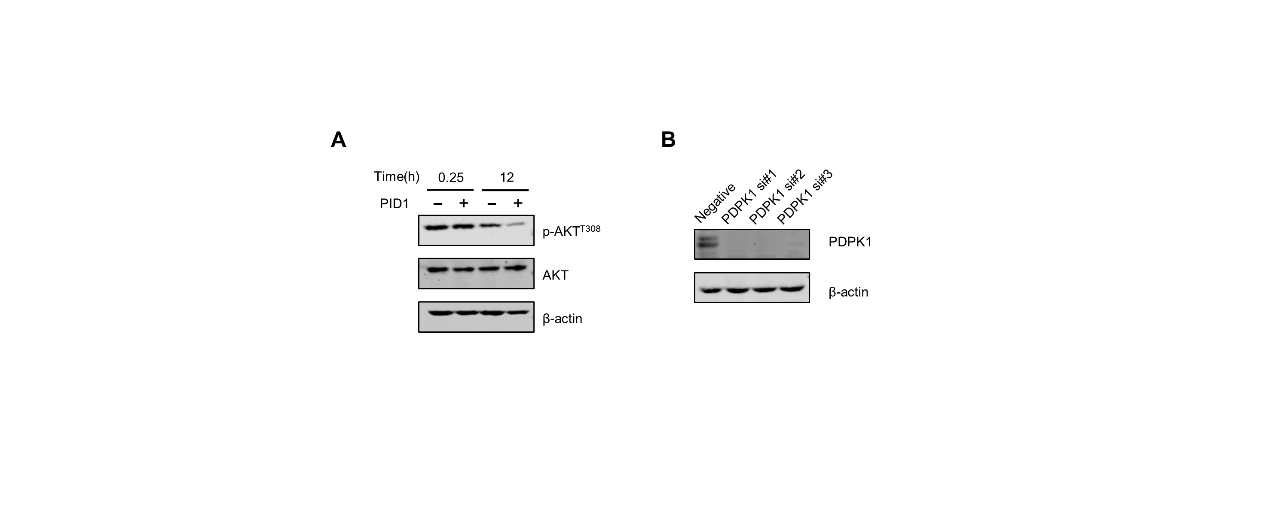


**Fig. S3 A** HepG2 cells with or without PID1 overexpression were treated with insulin (10 nM) for 0.25 h or 12 h, and protein levels of AKT, p-AKT^T308^ and β-actin were assessed by western blot analysis. **B** Western blot analysis of PDPK1 and β-actin in Hep3B cells with PDPK1 knockdown.


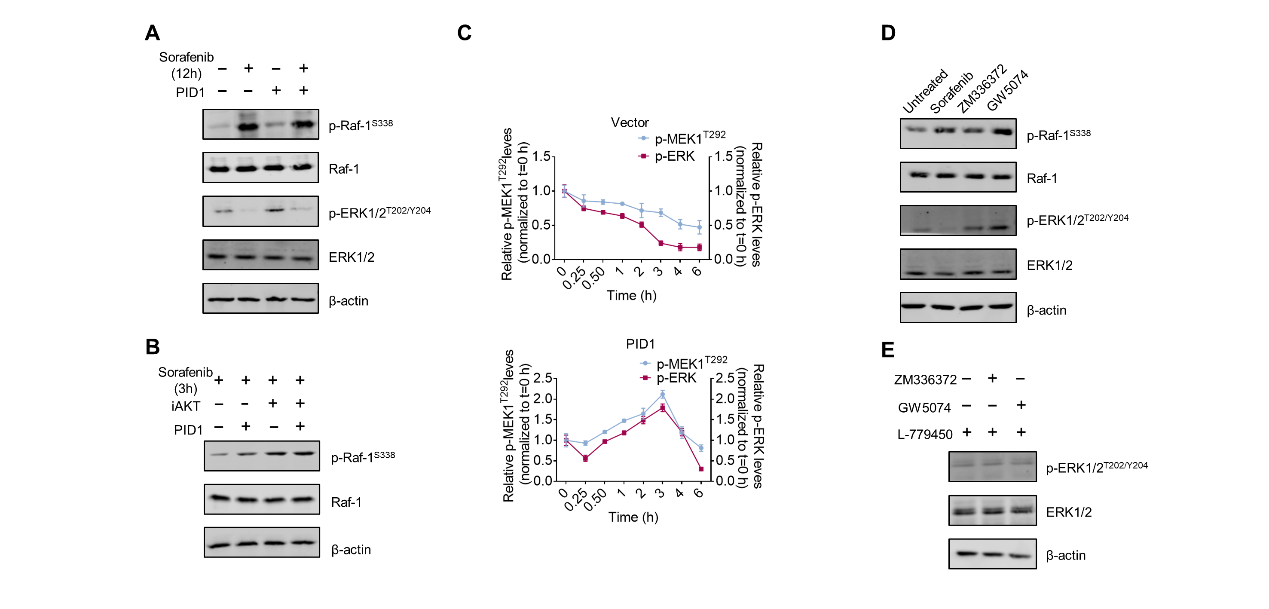


**Fig. S4 A** Western blot analysis of Raf-1, p-Raf-1^S338^, ERK1/2, p-ERK1/2^T202/Y204^ and β-actin in HepG2 cells with or without PID1 overexpression upon Sorafenib (10 μM) treatment for 12 h. **B** Western blot analysis of Raf-1, p-Raf-1^S338^ and β-actin in HepG2 cells with or without PID1 overexpression upon Sorafenib (10 μM) treatment for 12 h in the presence of AKT inhibitor VIII (10 μM). **C** Dynamics of MEK1 phosphorylation and ERK activation in HepG2 cells with or without PID1 overexpression upon Sorafenib (10 μM) treatment. p-MEK1^T292^ and p-ERK1/2^T202/Y204^ band density was normalized to actin and then normalized to t=0 h. **D** Western blot analysis of Raf-1, p-Raf-1^S338^, ERK1/2, p-ERK1/2^T202/Y204^ and β-actin in HepG2 cells treated with Sorafenib (10 μM), ZM336372 (20 μM) and GW5074 (10 μM) for 12 h. **E** Western blot analysis of ERK1/2, p-ERK1/2^T202/Y204^ and β-actin in HepG2 cells treated with ZM336372 (20 μM) or GW5074 (10 μM) for 12 h in the presence of L-779450 (20 μM).
