# Supplementary material for: Dual role of PID1 in regulating apoptosis induced by distinct anticancer-agents through AKT/Raf-1-dependent pathway in hepatocellular carcinoma
Source: Cell Death Discov. 2023 Apr 28;9:139. doi: 10.1038/s41420-023-01405-1 (PMC10147665; doi:10.1038/s41420-023-01405-1)

1 STR sites of Hep3B

| **Amelogenin** | X |
| --- | --- |
| **CSF1PO** | 8 |
| **D2S1338** | 21,25 |
| **D3S1358** | 15 |
| **D5S818** | 13 |
| **D7S820** | 8,10 |
| **D8S1179** | 12 |
| **D13S317** | 12,14 |
| **D16S539** | 10 |
| **D18S51** | 20 |
| **D19S433** | 12.2,14 |

| **D21S11** | 30,31 |
| --- | --- |
| **FGA** | 18 |
| **PentaD** | 12,14 |
| **PentaE** | 5,16 |
| **TH01** | 6,7 |
| **TPOX** | 9 |
| **vWA** | 17 |
| **D6S1043** | 12,17 |
| **D12S391** | 17 |
| **D2S441** | 10,12 |

STR profiling of Hep3B cells


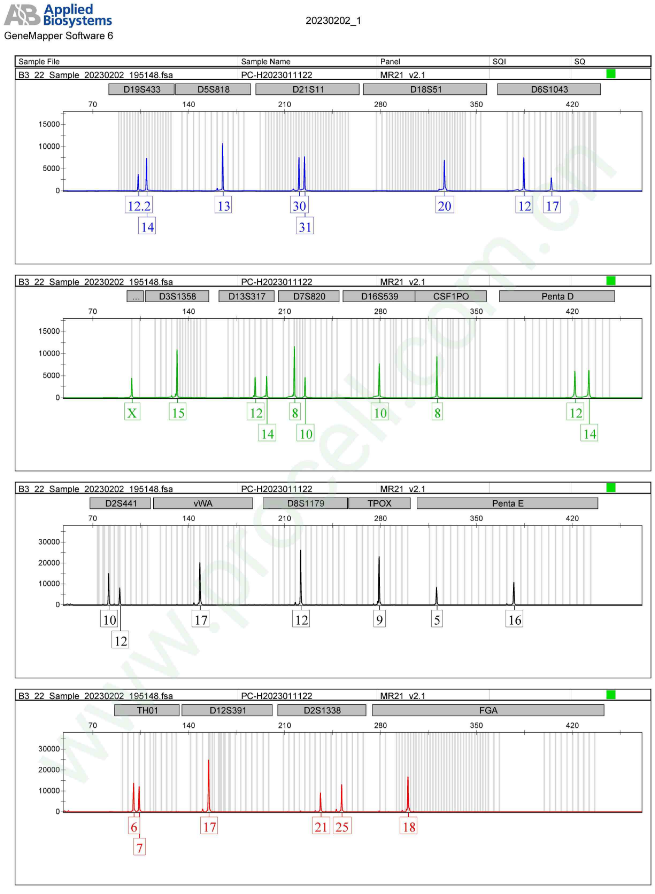


2 STR sites of HepG2

| **Amelogenin** | X |
| --- | --- |
| **CSF1PO** | 8 |
| **D2S1338** | 21,25 |
| **D3S1358** | 15 |
| **D5S818** | 13 |
| **D7S820** | 8,10 |
| **D8S1179** | 12 |
| **D13S317** | 12,14 |
| **D16S539** | 10 |
| **D18S51** | 20 |
| **D19S433** | 12.2,14 |

| **D21S11** | 30,31 |
| --- | --- |
| **FGA** | 18 |
| **PentaD** | 12,14 |
| **PentaE** | 5,16 |
| **TH01** | 6,7 |
| **TPOX** | 9 |
| **vWA** | 17 |
| **D6S1043** | 12,17 |
| **D12S391** | 17 |
| **D2S441** | 10,12 |

STR profiling of HepG2 cells


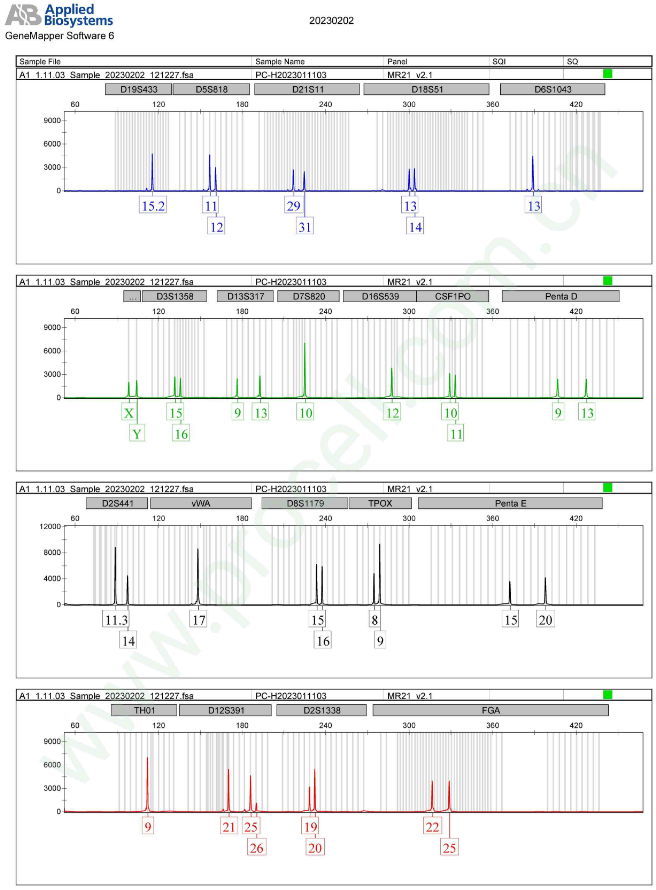


3 STR sites of SK-Hep-1

| **Amelogenin** | X |  |
| --- | --- | --- |
| **CSF1PO** | 11,12 |  |
| **D2S1338** | 20,23 |  |
| **D3S1358** | 16 |  |
| **D5S818** | 10,13 |  |
| **D7S820** | 8,11 |  |
| **D8S1179** | 13,14 |  |
| **D13S317** | 8,12 |  |
| **D16S539** | 12 |  |
| **D18S51** | 13,15 |  |
| **D19S433** | 12,15.2 |  |

| **D21S11** | 29,31 |
| --- | --- |
| **FGA** | 17 |
| **PentaD** | 13,14 |
| **PentaE** | 13,21 |
| **TH01** | 7,9 |
| **TPOX** | 9 |
| **vWA** | 14,17 |
| **D6S1043** | 11 |
| **D12S391** | 18,19 |
| **D2S441** | 12.3,14 |

STR profiling of SK-Hep-1 cells


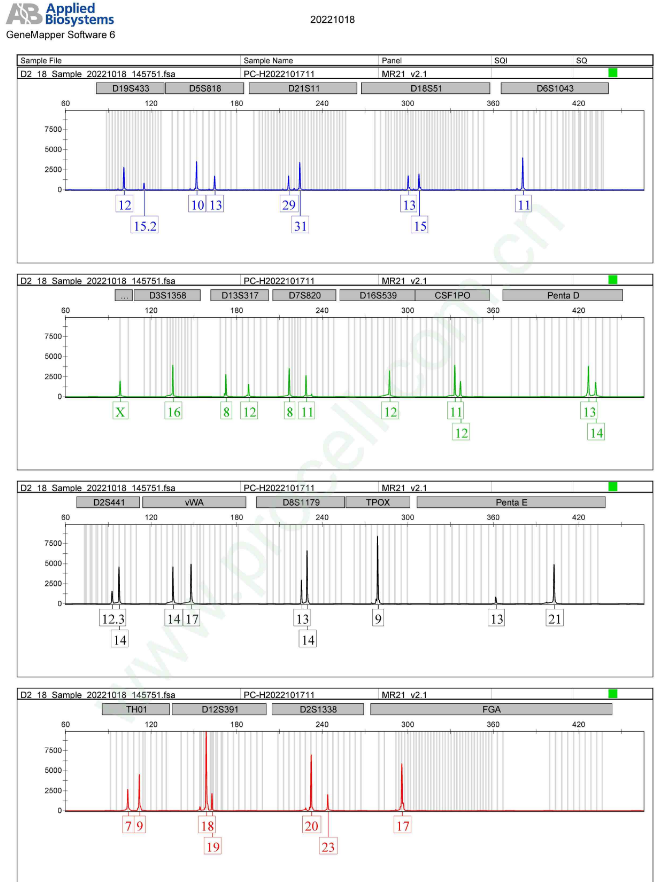

Supplement: Supplementary file 2 — STR sites information [file 41420_2023_1405_MOESM2_ESM.docx]
