# Supplementary material for: Dual role of PID1 in regulating apoptosis induced by distinct anticancer-agents through AKT/Raf-1-dependent pathway in hepatocellular carcinoma
Source: Cell Death Discov. 2023 Apr 28;9:139. doi: 10.1038/s41420-023-01405-1 (PMC10147665; doi:10.1038/s41420-023-01405-1)

## Figure 1A

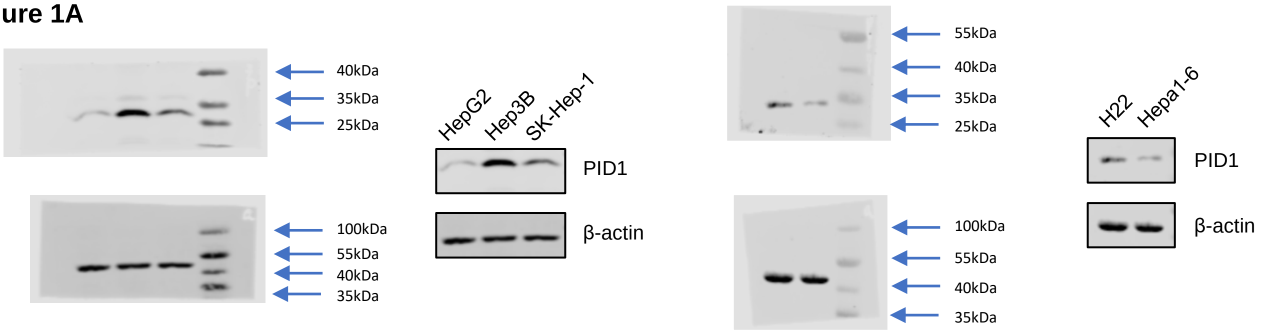


## Figure 3B

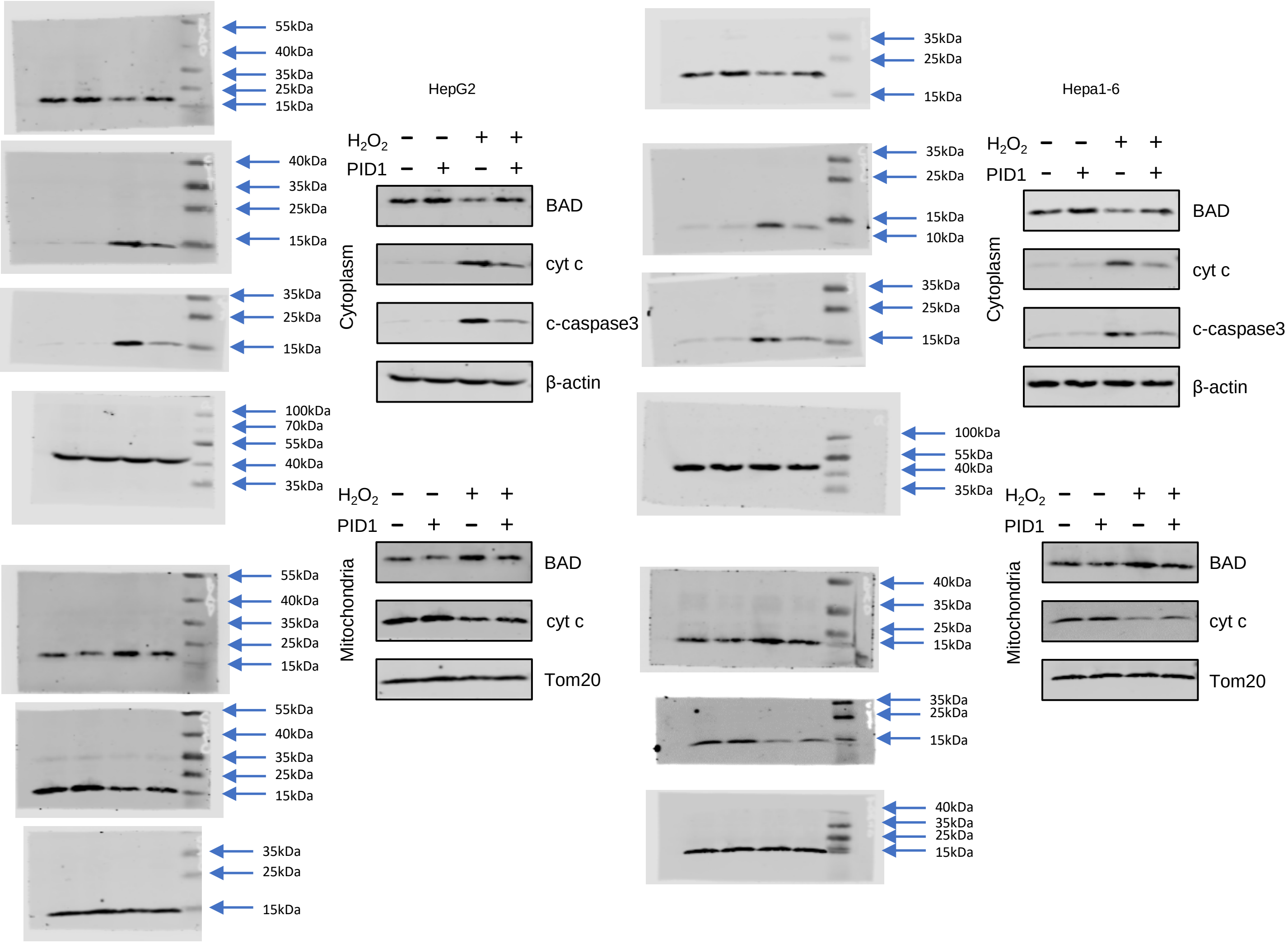

# Figure 3E

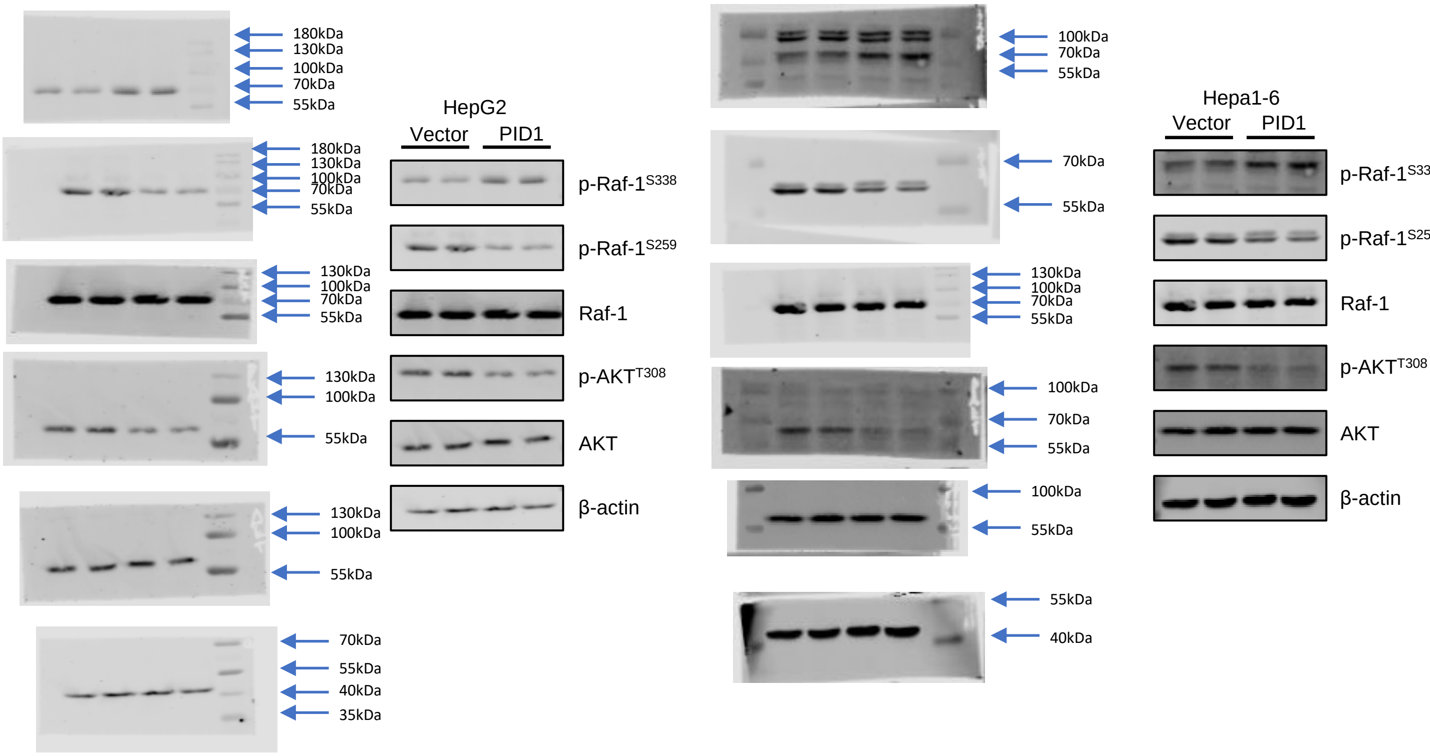
180kDa
130kDa
100kDa
70kDa
55kDa
40kDa
35kDa
HepG2
Vector
PID1
p-Raf-1S338
p-Raf-1S259
Raf-1
p-AKTT308
AKT
β-actin
Hepa1-6
Vector
PID1
Raf-1
p-AKTT308
AKT
β-actin

## Figure 3F

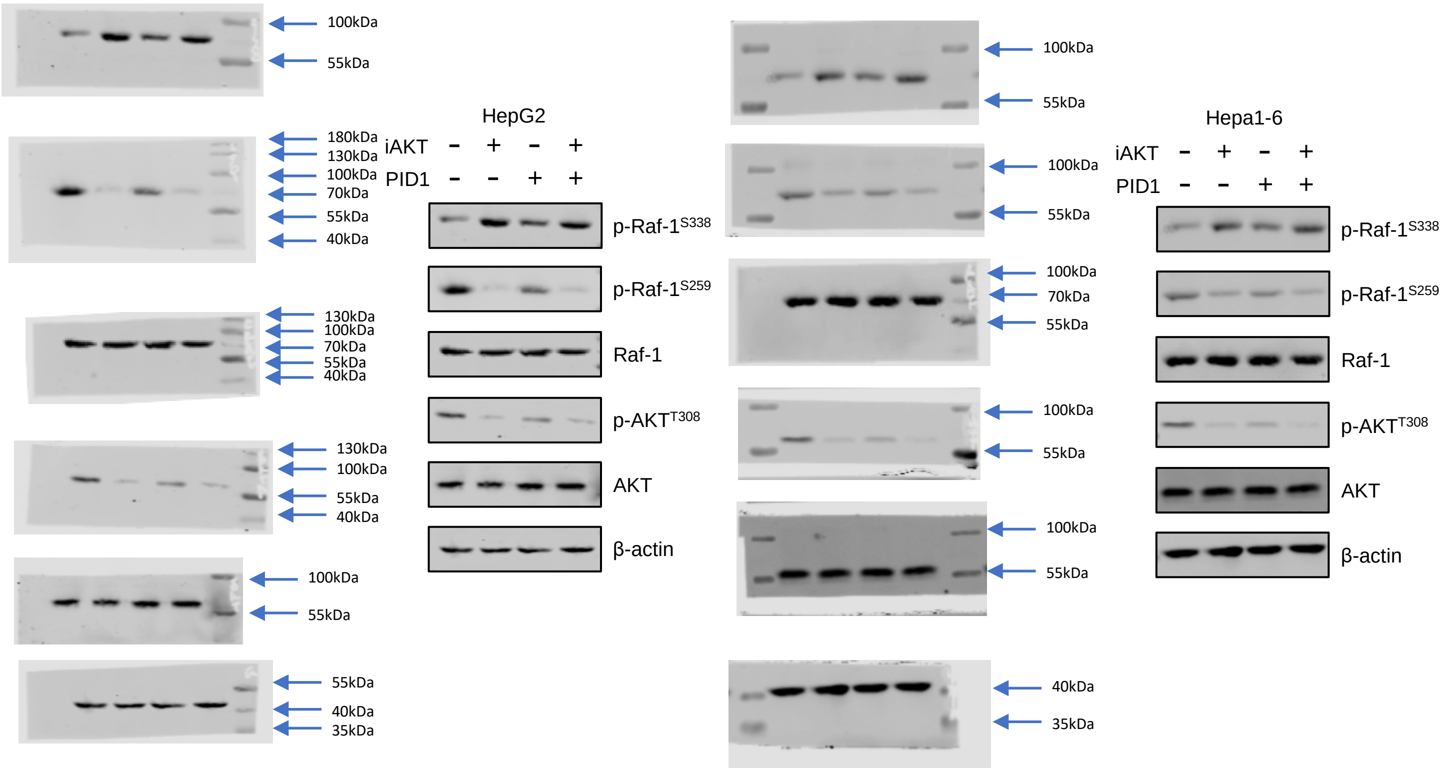


## Figure 3J

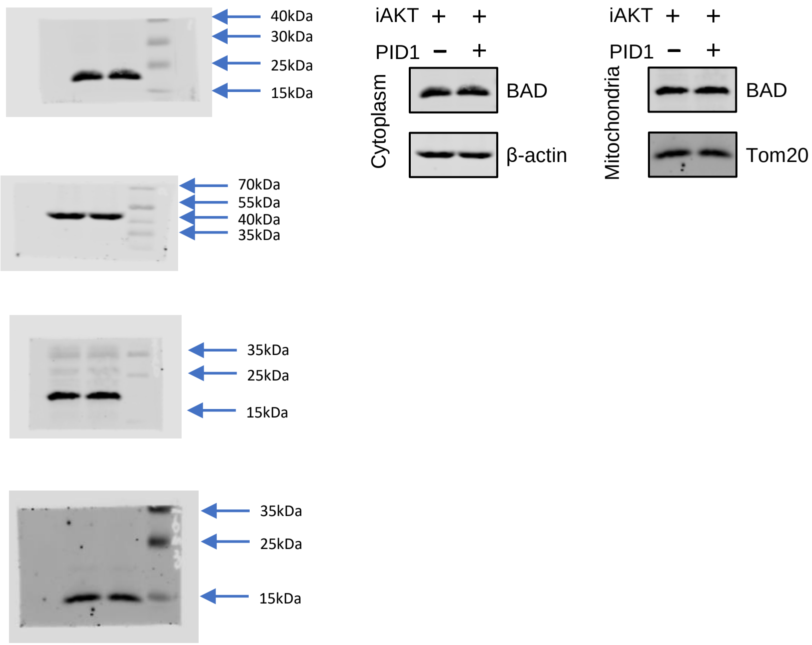


## Figure 3K

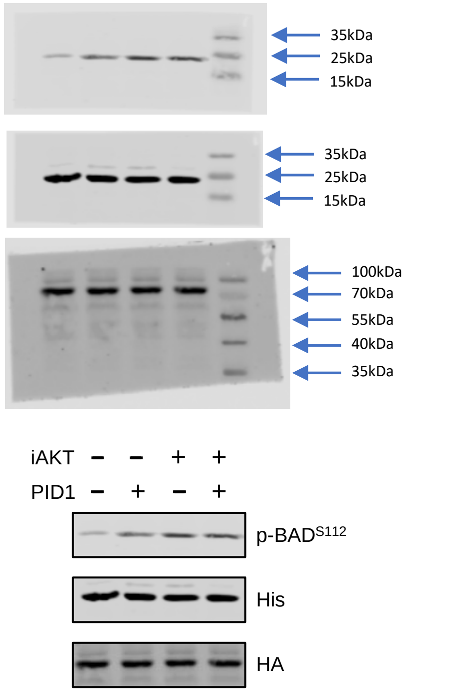

**Figure 4A**

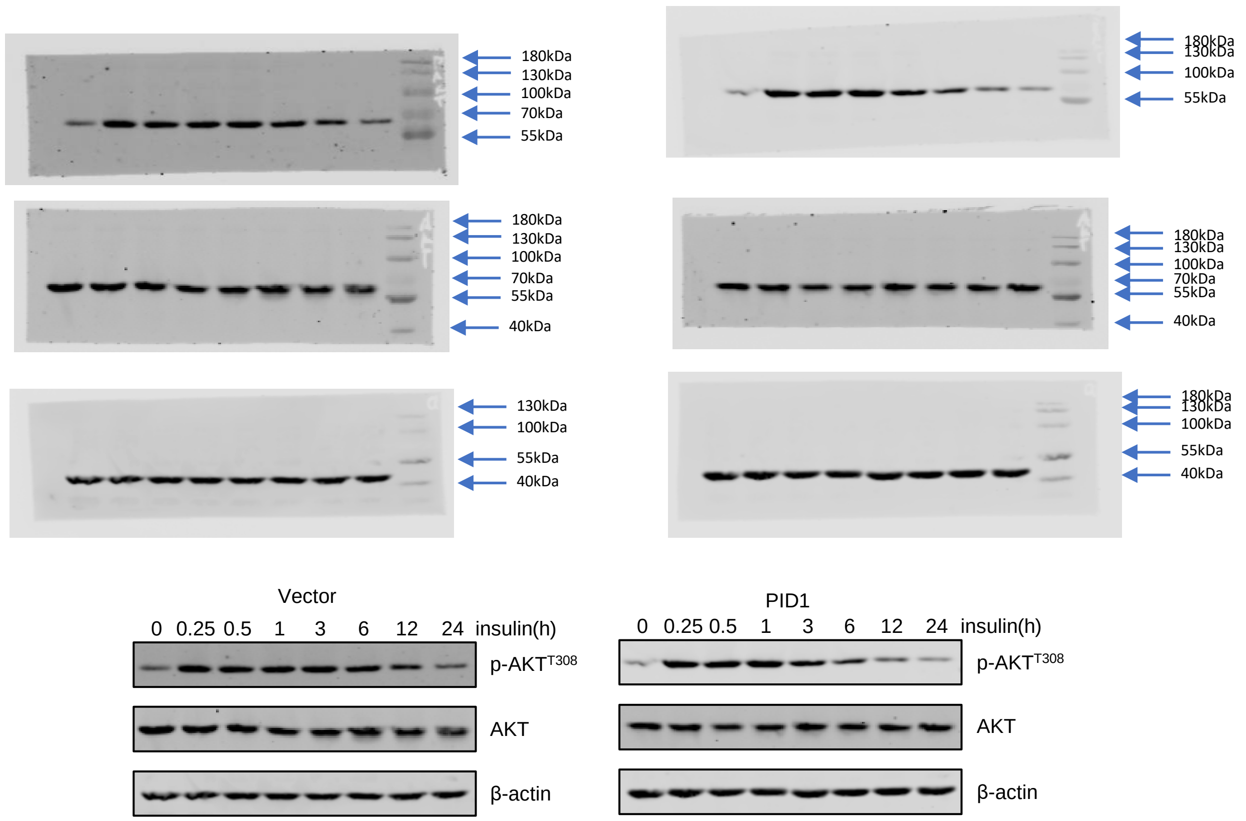
180kDa
130kDa
100kDa
70kDa
55kDa
40kDa
Vector
PID1
0 0.25 0.5 1 3 6 12 24 insulin(h)
p-AKT^T308
AKT
β-actin

**Figure 4B**

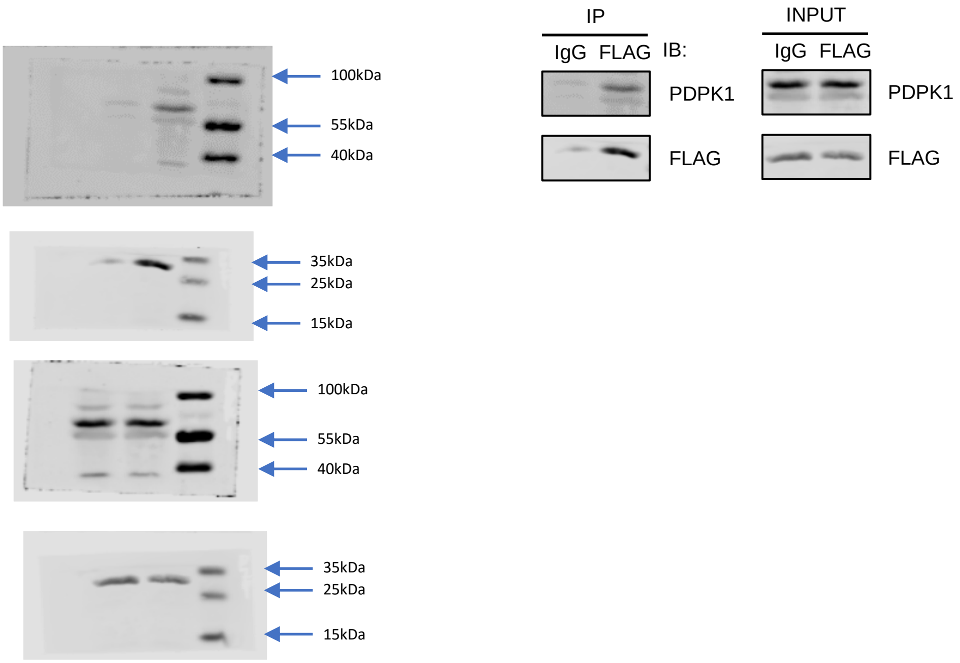


**Figure 4C**

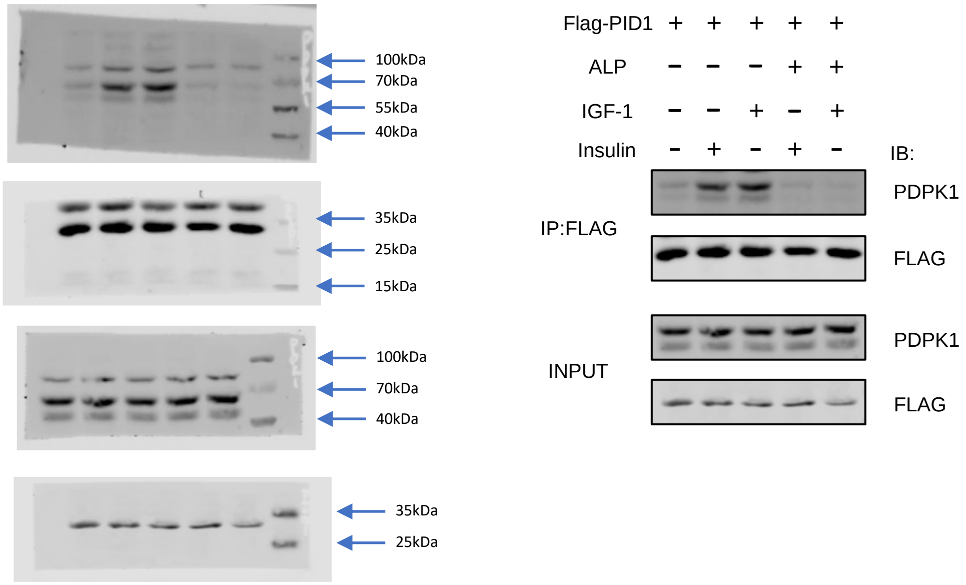

## Figure 4D

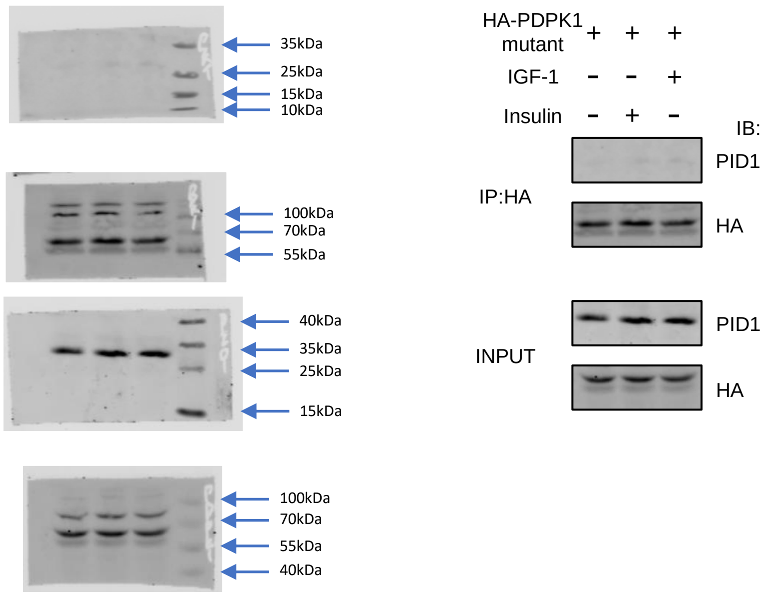


## Figure 4E

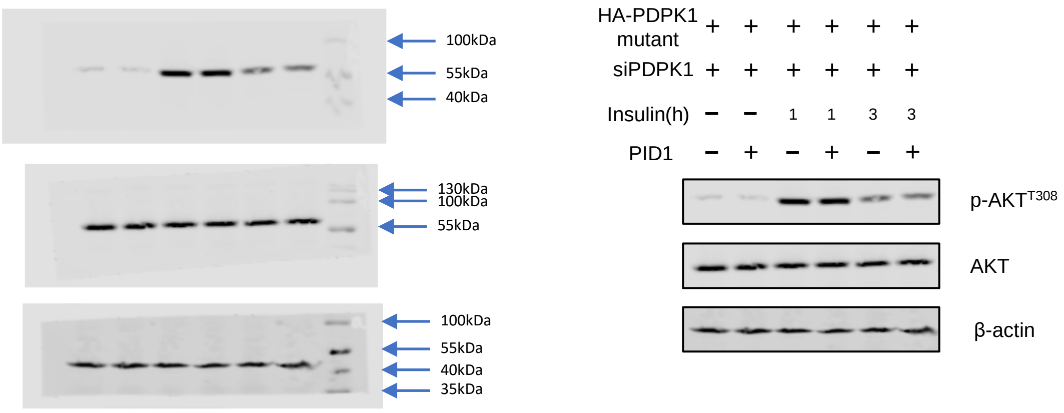

## Figure 5A

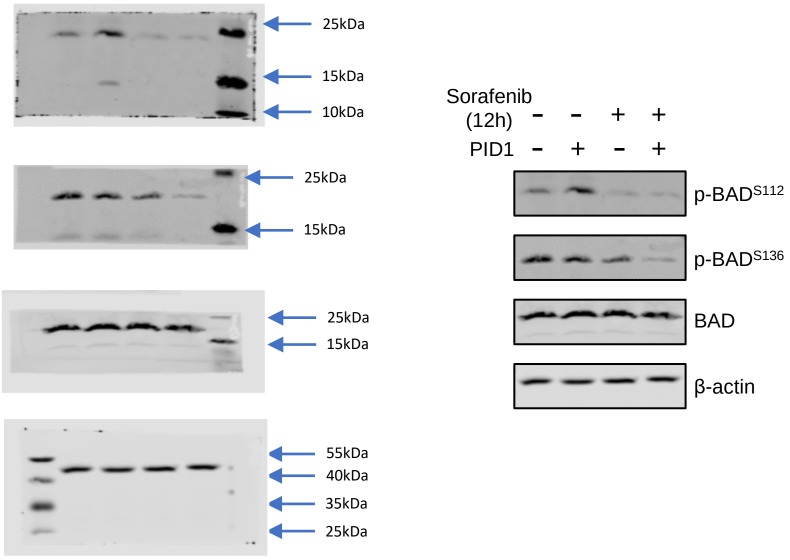


## Figure 5B

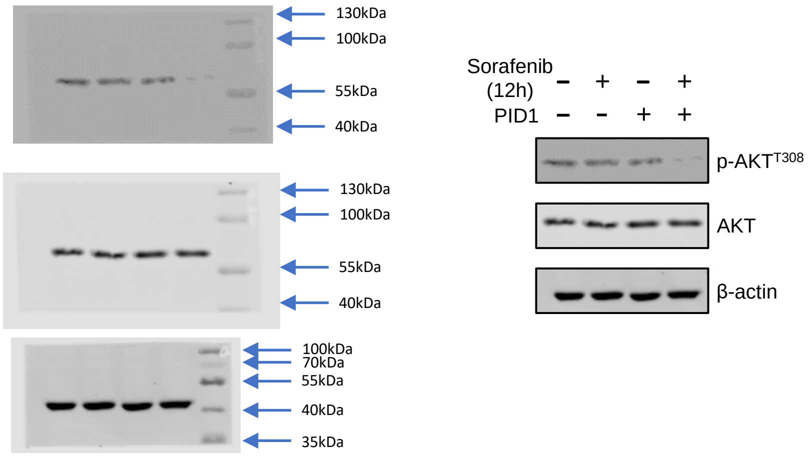

**Figure 5C**

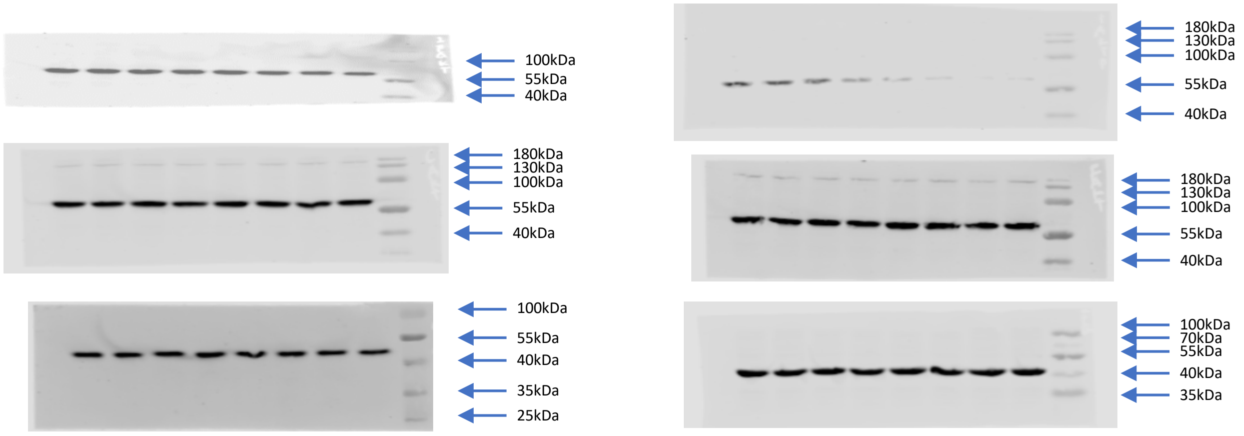


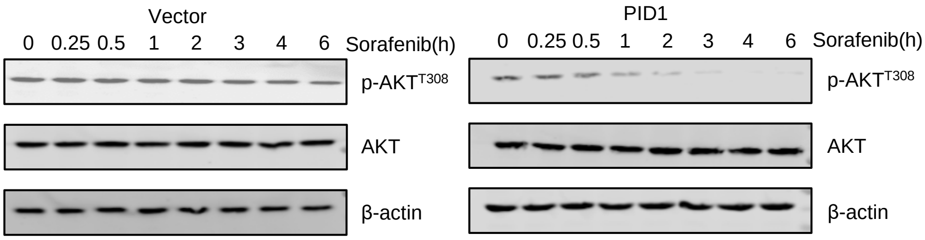


**Figure 5D**

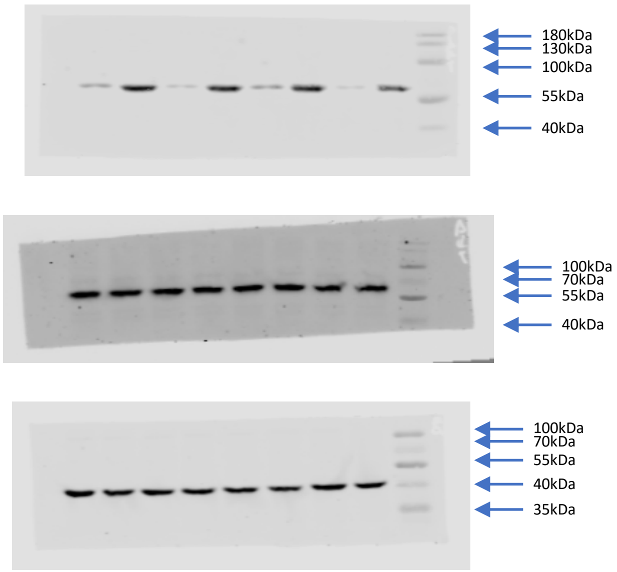


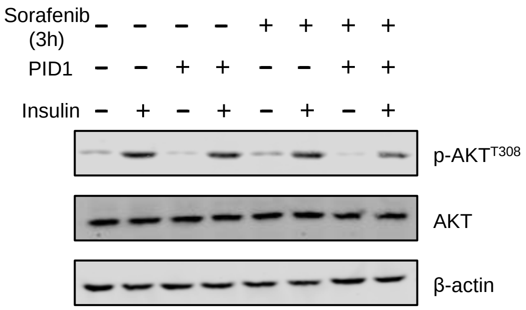

**Figure 5E**

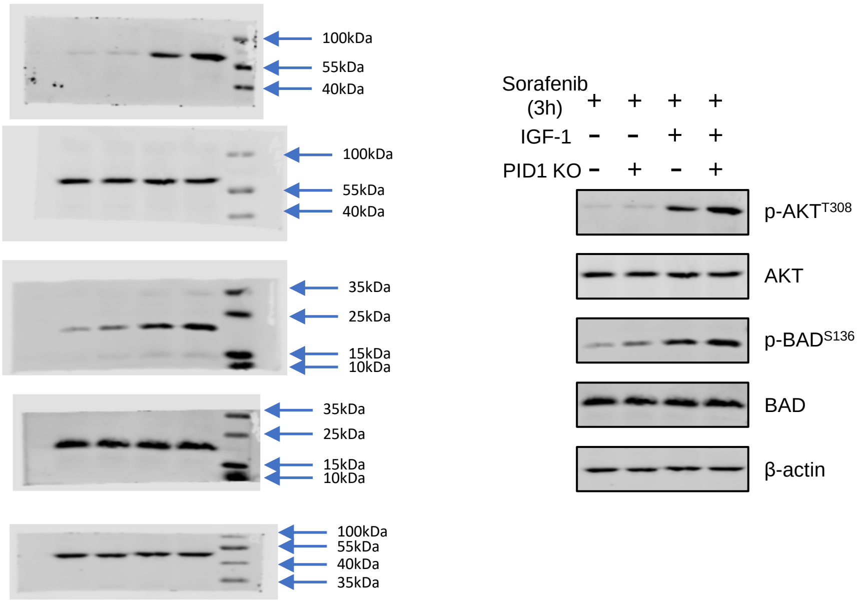

100kDa
55kDa
40kDa
100kDa
55kDa
40kDa
35kDa
25kDa
15kDa
10kDa
35kDa
25kDa
15kDa
10kDa
100kDa
55kDa
40kDa
35kDa
Sorafenib (3h) + + + +
IGF-1 – – + +
PID1 KO – + – +
p-AKTT308
AKT
p-BADS136
BAD
β-actin

**Figure 6A**

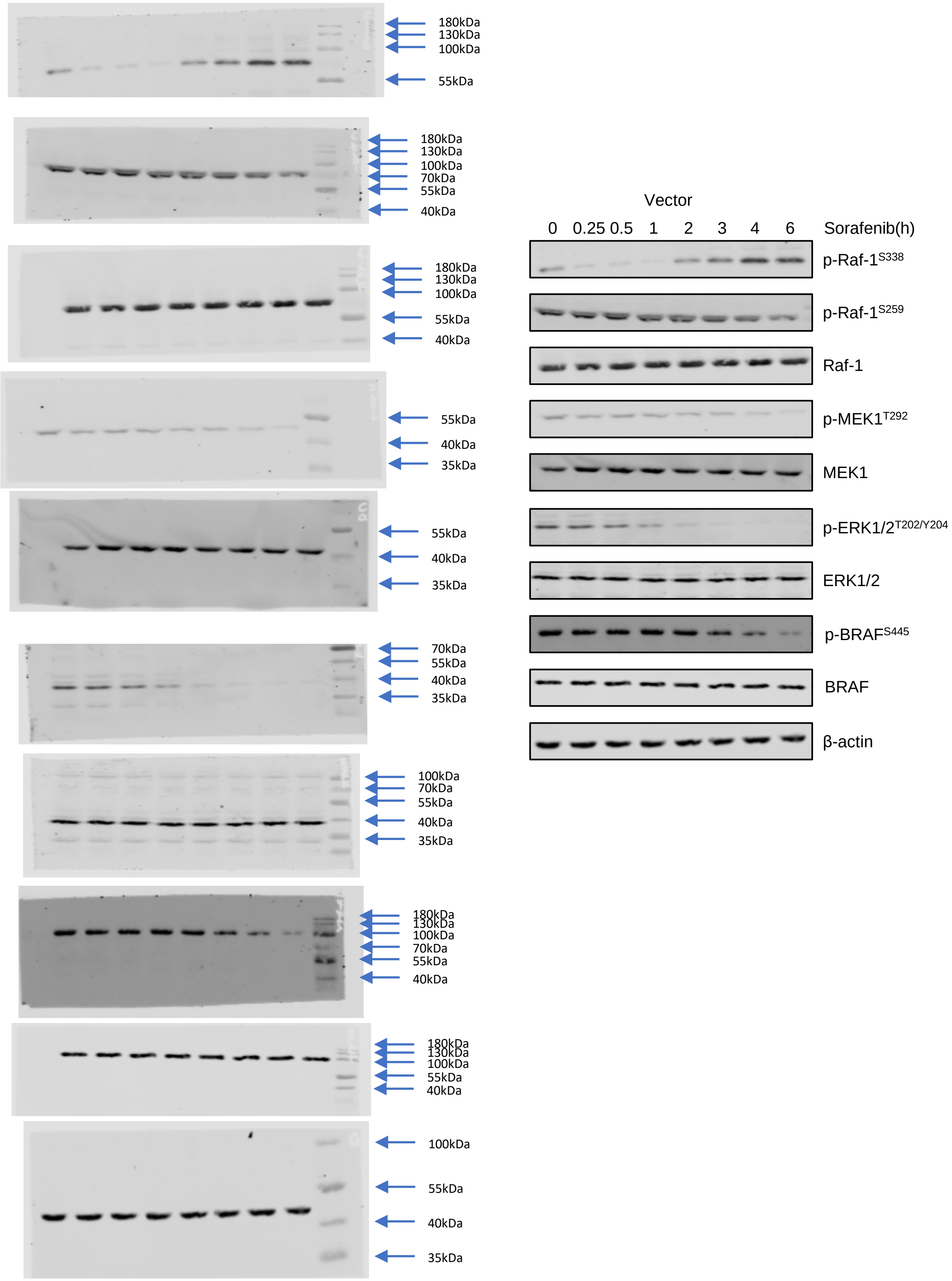
180kDa
130kDa
100kDa
55kDa
180kDa
130kDa
100kDa
70kDa
55kDa
40kDa
180kDa
130kDa
100kDa
55kDa
40kDa
55kDa
40kDa
35kDa
55kDa
40kDa
35kDa
70kDa
55kDa
40kDa
35kDa
100kDa
70kDa
55kDa
40kDa
35kDa
180kDa
130kDa
100kDa
70kDa
55kDa
40kDa
180kDa
130kDa
100kDa
55kDa
40kDa
100kDa
55kDa
40kDa
35kDa
Vector
0
0.25
0.5
1
2
3
4
6
Sorafenib(h)
p-Raf-1S338
p-Raf-1S259
Raf-1
p-MEK1T292
MEK1
p-ERK1/2T202/Y204
ERK1/2
p-BRAFS445
BRAF
β-actin

**Figure 6A**

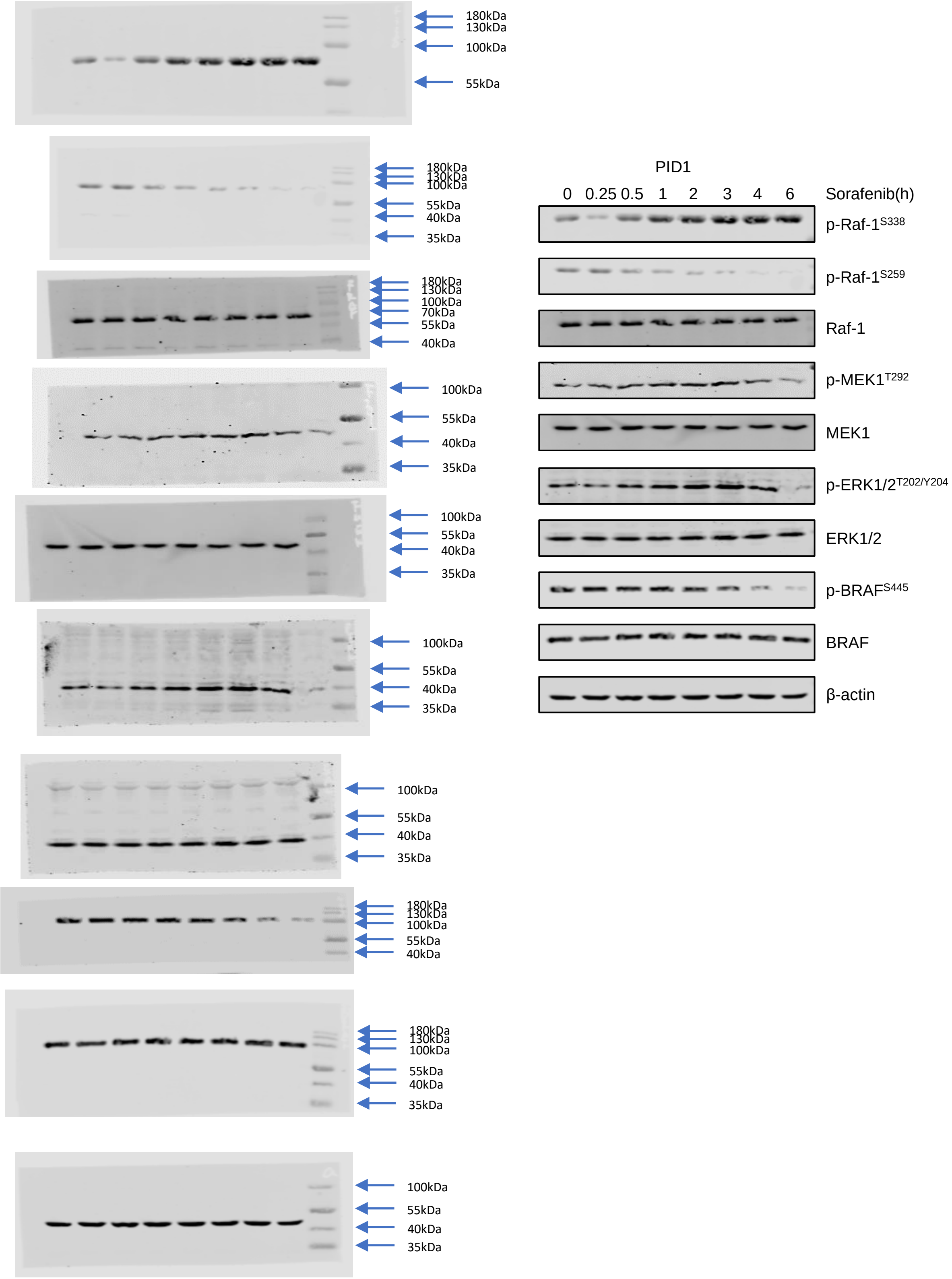
180kDa
130kDa
100kDa
70kDa
55kDa
40kDa
35kDa
PID1
0 0.25 0.5 1 2 3 4 6
Sorafenib(h)
p-Raf-1S338
p-Raf-1S259
Raf-1
p-MEK1T292
MEK1
p-ERK1/2T202/Y204
ERK1/2
p-BRAFS445
BRAF
β-actin

## Figure 6C

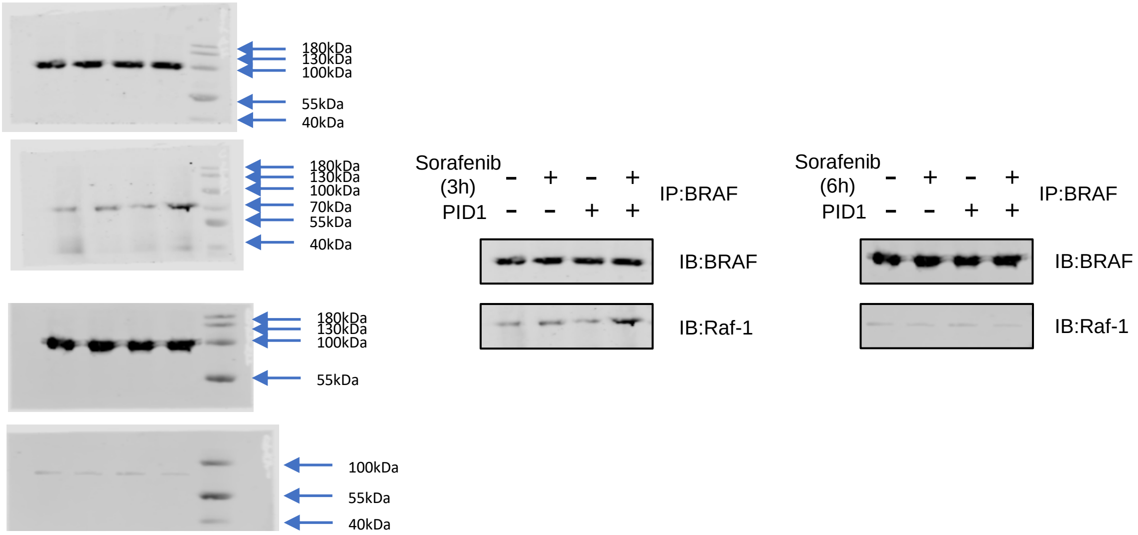


## Figure 6E

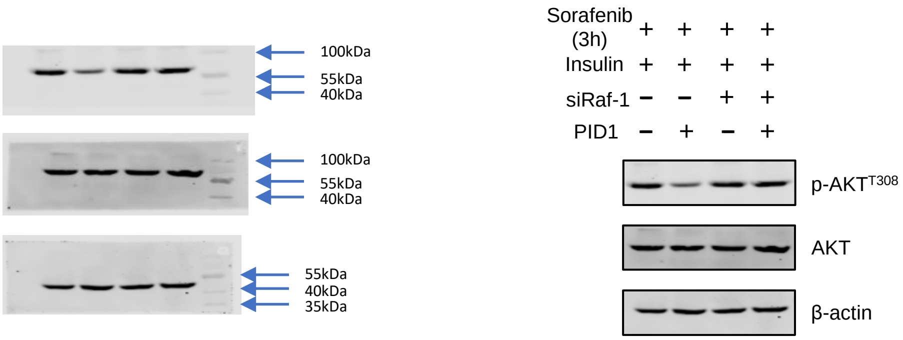

# Figure 6D

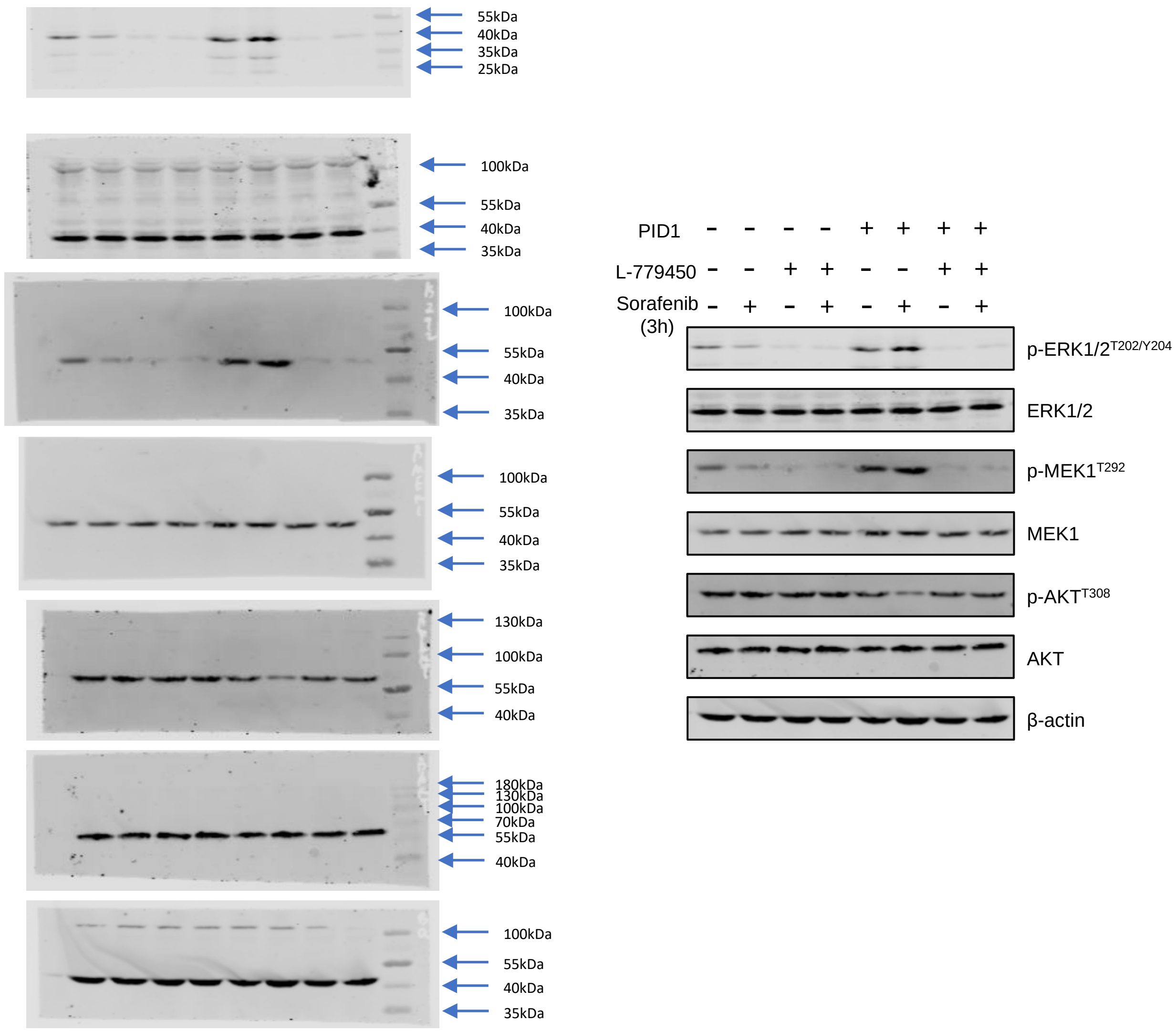

55kDa
40kDa
35kDa
25kDa
100kDa
55kDa
40kDa
35kDa
100kDa
55kDa
40kDa
35kDa
100kDa
55kDa
40kDa
35kDa
130kDa
100kDa
55kDa
40kDa
180kDa
130kDa
100kDa
70kDa
55kDa
40kDa
100kDa
55kDa
40kDa
35kDa
PID1 – – – – + + + +
L-779450 – – + + – – + +
Sorafenib (3h) – + – + – + – +
p-ERK1/2T202/Y204
ERK1/2
p-MEK1T292
MEK1
p-AKTT308
AKT
β-actin

## Fig. S1A

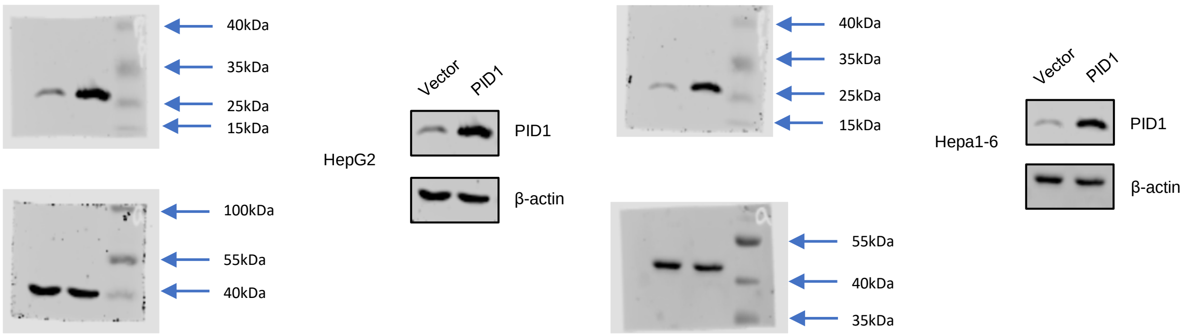


## Fig. S1E

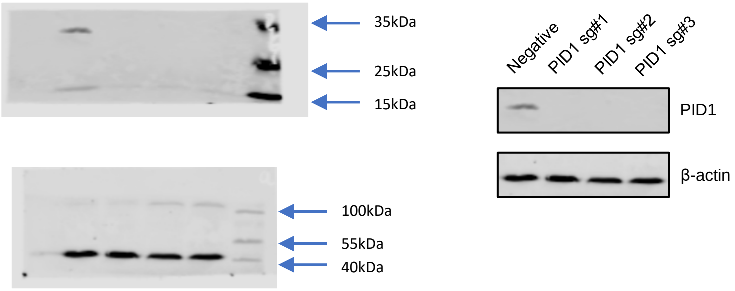

## Fig. S2A

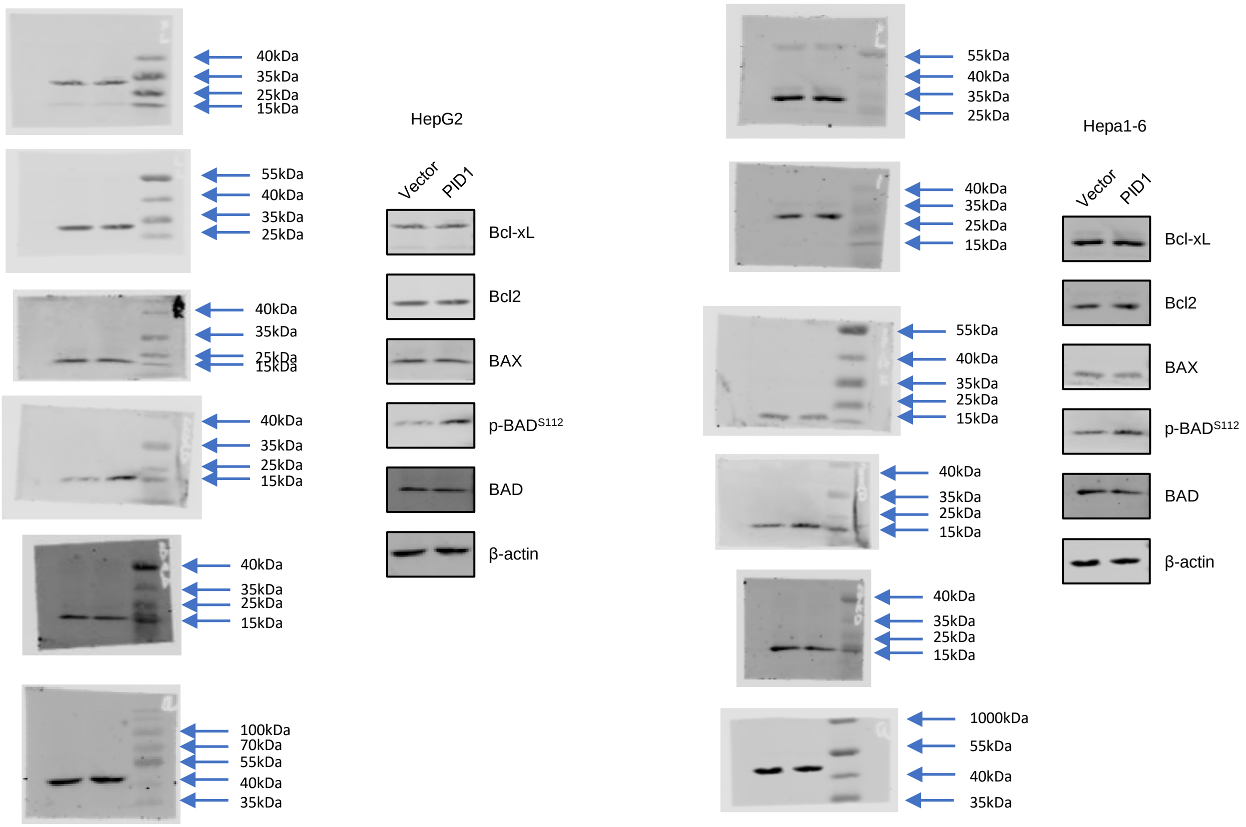


## Fig. S2F

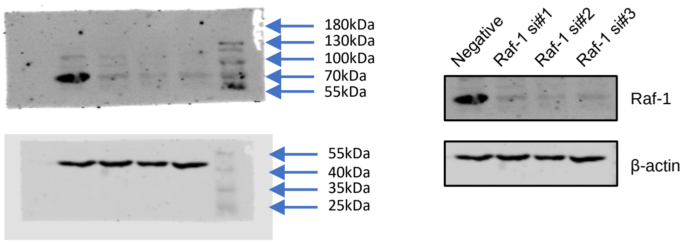

## Fig. S3A

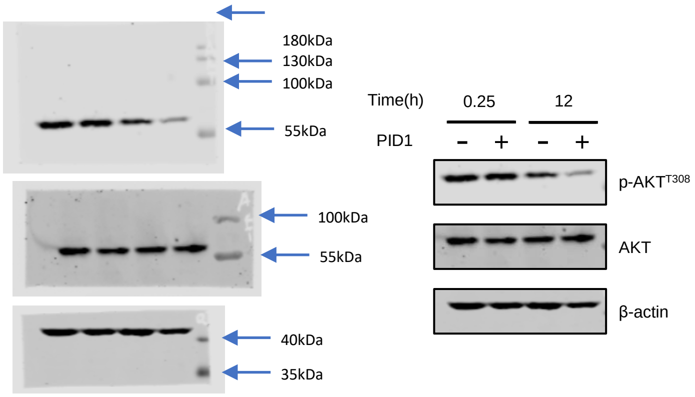


## Fig. S3B

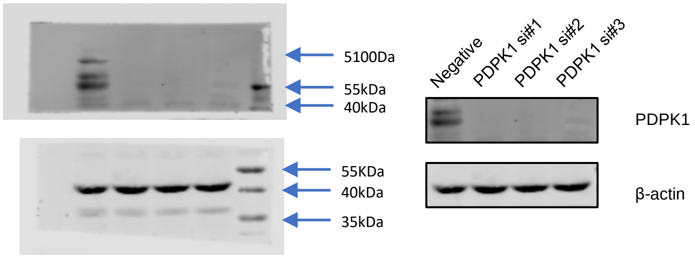

Fig. S4A

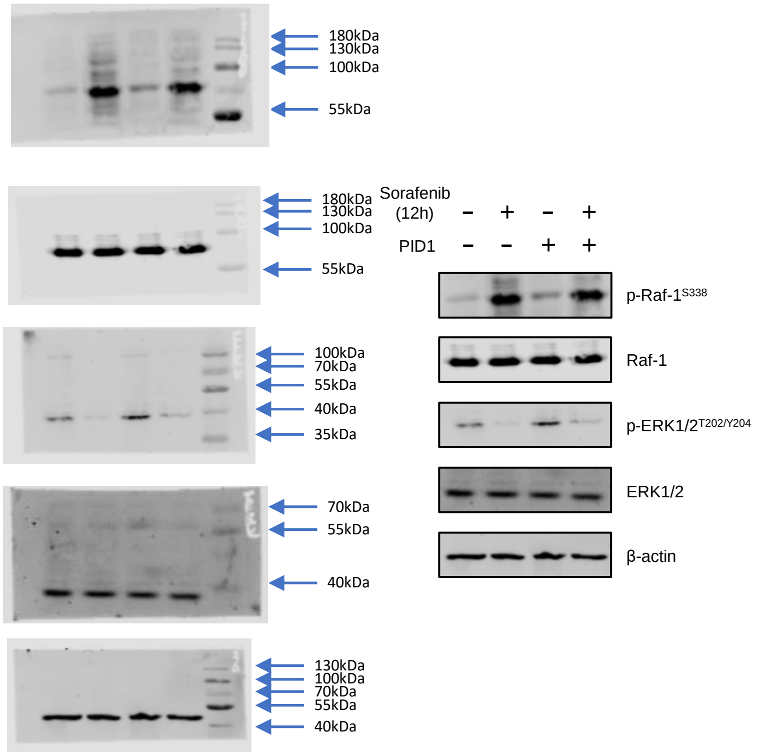


Fig. S4B

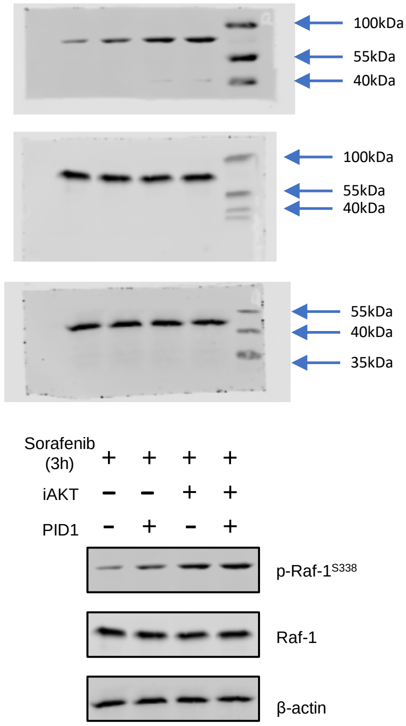


Fig. S4D

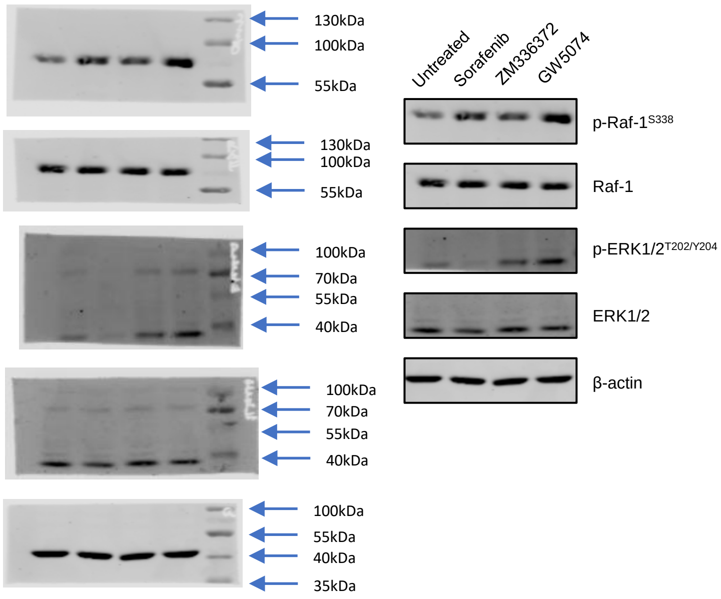


Fig. S4E

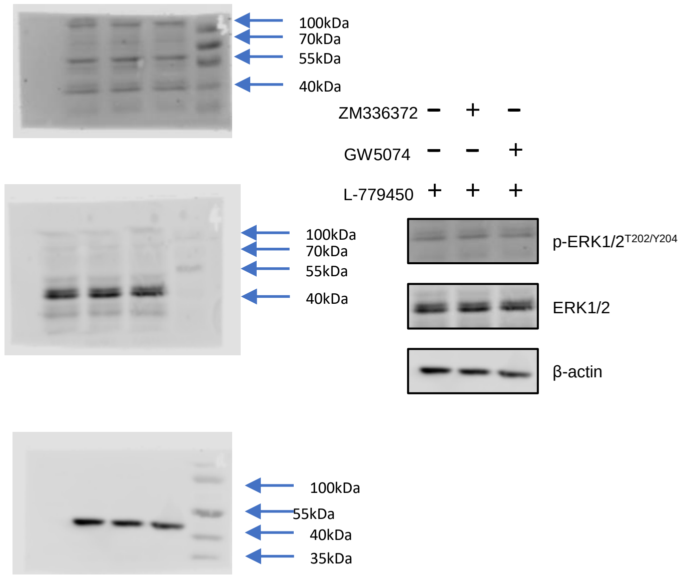

Supplement: Supplementary file 3 — Original Data File [file 41420_2023_1405_MOESM3_ESM.pdf]
